# Molecular strategy for the direct detection and identification of human enteroviruses in clinical specimens associated with hand, foot and mouth disease

**DOI:** 10.1371/journal.pone.0241614

**Published:** 2020-11-09

**Authors:** Yonghong Zhou, Qi Qiu, Kaiwei Luo, Qiaohong Liao, Yu Li, Peng Cui, Lu Liang, Yibing Cheng, Lili Wang, Kai Wang, Le Van Tan, H. Rogier van Doorn, Hongjie Yu

**Affiliations:** 1 School of Public Health, Fudan University, Key Laboratory of Public Health Safety, Ministry of Education, Shanghai, China; 2 Hunan Provincial Center for Disease Control and Prevention, Changsha, Hunan Province, China; 3 Division of Infectious Disease, Key Laboratory of Surveillance and Early-warning on Infectious Disease, Chinese Centre for Disease Control and Prevention, Beijing, China; 4 West China School of Public Health, Sichuan University, Chengdu, China; 5 Hospital Affiliated to Zhengzhou University, Henan Children’s Hospital, Zhengzhou, China; 6 Oxford University Clinical Research Unit, Ho Chi Minh City, Vietnam; 7 Centre for Tropical Medicine and Global Health, Nuffield Department of Medicine, University of Oxford, Oxford, United Kingdom; Institut Pasteur, FRANCE

## Abstract

**Background:**

Diseases caused by human enteroviruses (EVs) are a major global public health problem. Thus, the effective diagnosis of all human EVs infections and the monitoring of epidemiological and ecological dynamic changes are urgently needed.

**Methods:**

Based on two comprehensive virological surveillance systems of hand, foot and mouth disease (HFMD), real-time PCR and nested RT-PCR (RT-snPCR) methods based on the enteroviral VP1, VP4-VP2 and VP4 regions were designed to directly detect all human EVs serotypes in clinical specimens.

**Results:**

The results showed that the proposed serotyping strategy exhibit very high diagnostic efficiency (Study 1: 99.9%; Study 2: 89.5%), and the variance between the study was due to inclusion of the specific Coxsackie virus A6 (CVA6) real-time RT-PCR and VP4 RT-snPCR in Study 1 but not Study 2. Furthermore, only throat swabs were collected and analyzed in Study 2, whereas in Study 1, if a specific EV serotype was not identified in the primary stool sample, other sample types (rectal swab and throat swab) were further tested where available. During the study period from 2013 to 2018, CVA6 became one of the main HFMD causative agents, whereas the level of enterovirus A71 (EV-A71) declined in 2017.

**Conclusion:**

The findings of this study demonstrate the appropriate application of PCR methods and the combination of biological sample types that are useful for etiological studies and propose a molecular strategy for the direct detection of human EVs in clinical specimens associated with HFMD.

## Introduction

Infectious diseases caused by human enteroviruses (EVs) are a major, global public health problem [1, 2]. Although infections with human EVs are common and mostly asymptomatic, severe infections, involving symptoms of the central nervous system for example, can be fatal [3].

*Enterovirus*, which is a genus within the *Picornaviridae* family [4], consists of 15 species, namely, *enteroviruses* A-L and *rhinoviruses* A-C, and more than 300 serotypes (http://www.picornaviridae.com/enterovirus/enterovirus.htm). EVs are nonenveloped, single-stranded, positive-sense, polyadenylated RNA viruses, and their viral genome is 7 and 8.8 kb in length [3].

Hand, foot, and mouth disease (HFMD) is mainly caused by human EVs species A (HEV-A) [5], and it is sometimes difficult to distinguish HFMD from other rash diseases, such as chickenpox, impetigo, and measles [6–11]. In addition, the clinical manifestation of severe HFMD with CNS involvement might be similar to that of herpes simplex virus, cytomegalovirus (CMV) and Epstein–Barr virus (EBV) induced encephalitis or meningitis [11]. The etiological agents of HFMD cannot be deduced based only on the clinical picture [11]. The other reasons for the difficulty in distinguishing HFMD include the similarity of the symptoms caused by different serotypes, and the rapid evolution of RNA viruses, which results in high EVs diversity and cocirculation, frequent recombination and viral natural selection. Furthermore, specific serotypes, such as EV-A71, might be associated with a greater probability of unfavorable outcomes. The Chinese Guidelines for the Diagnosis and Treatment of Hand, Foot and Mouth Disease (2018 Edition) showed that the early recognition of severe cases is of utmost importance in the diagnosis and treatment of patients with HFMD [11]. To reduce risk, improve the diagnostic specificity and subsequently enhance the management of patient, a strategy for the detection and typing of EVs that is reliable, convenient and fast would be of great value to front-line physicians [12].

Vaccination against HFMD enterovirus A71 (EV-A71) was implemented in China in March 2016 [13]. However, the incidence of HFMD in China has not declined, and this disease still ranks first among the national legally reported infectious diseases [14]. Due to the introduction of this vaccine, a shift in the main causative EVs serotypes and/or genetic characteristics and transmission patterns might be expected in subsequent years. In addition, the national HFMD surveillance system showed that non-EV-A71 and non-Coxsackie virus A16 (non-CVA16) EVs became the main epidemic strains in 2013, 2015 and 2017 [15–17]. Therefore, in this postvaccine era, an EV detection and typing strategy is crucial for supporting continuous comprehensive virological surveillance, which would allow the identification of epidemiological and ecological changes in the HFMD dynamics.

At present, the gold standard diagnostic method for EVs infections require reference laboratory confirmation is virus isolation. This method is time- and lab-resource- intensive and prone to serological cross-reactivity and exhibit limited sensitivity. Most importantly, due to the long delay from sample collection to results, these methods provide little value to informing clinical treatment [4, 12, 18]. However, molecular detection methods have rapidly developed over the last 30 years, and these provide specific and sensitive approaches for EVs diagnosis [18, 19]. Based on the sequences and antigenicity of four enteroviral structural proteins (VP1–VP4), EVs can be divided into different serotypes/genotypes, and as a result, the current VP1 typing approaches have become the accepted “gold standard” for EVs typing [18].

Despite their increased sensitivity for the identification of EVs, a single PCR remains unable to identify all serotypes in a single clinical sample. The existing universal primers show marked variations in sensitivity and specificity, particularly if the clinical specimen has a low viral load [20, 21]. For example, the study conducted by Nix, et al. showed that a VP1 nested RT-PCR (RT-snPCR) assay can identify all EVs directly from any clinical specimen [22]. However, in our previous study, we used generic real-time PCR methods in combination with this VP1 RT-snPCR for EVs detection and found that 4% of samples (116 of 2836) could not be serotyped [23].

In this study, we proposed a molecular strategy for the detection of all EVs serotypes from clinical samples. A real-time RT-PCR combined with several RT-PCR methods, based on the enteroviral VP1 and VP4-VP2 regions was designed to directly detect and speciate all human EVs in clinical specimens associated with HFMD.

## Materials and methods

### Data sources

The clinical specimens originated from two comprehensive virological surveillance studies of HFMD performed in northern (Zhengzhou City, Henan Province) and southern (Anhua County, Hunan Province) China.

Study 1: All patients aged ≤ 14 years who were hospitalized with HFMD at any of the six participating hospitals in Anhua County between October 1, 2013, and September 30, 2016, were enrolled, and throat swabs, rectal swabs or stool samples were collected [23].

Study 2: Three outpatient HFMD cases at Zhengzhou Children’s Hospital in Zhengzhou City were enrolled every two days between February 15, 2017, and April 4, 2018 and throat swabs were collected [24].

### Laboratory testing

**Fig 1:**
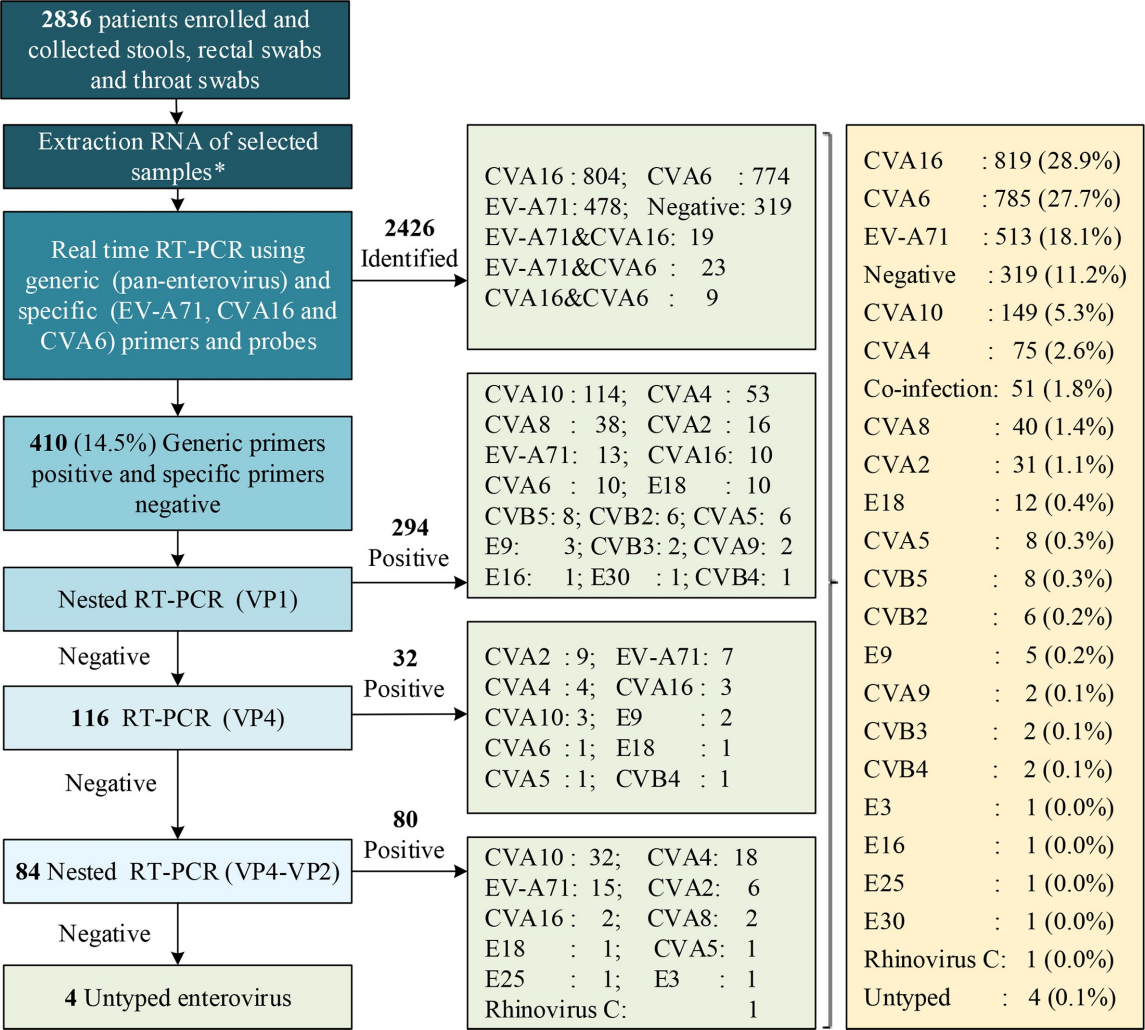
Overview of the testing assay procedures and typing results in Anhua County (Study 1). N.B: “*” indicates that the clinical samples satisfied the ideal selection criteria for typing, and the stool samples were selected first. If the stool samples were negative or untyped, rectal swabs were used for typing, and it the rectal swabs also yielded negative results, throat swabs were used for typing if available.

**Fig 2:**
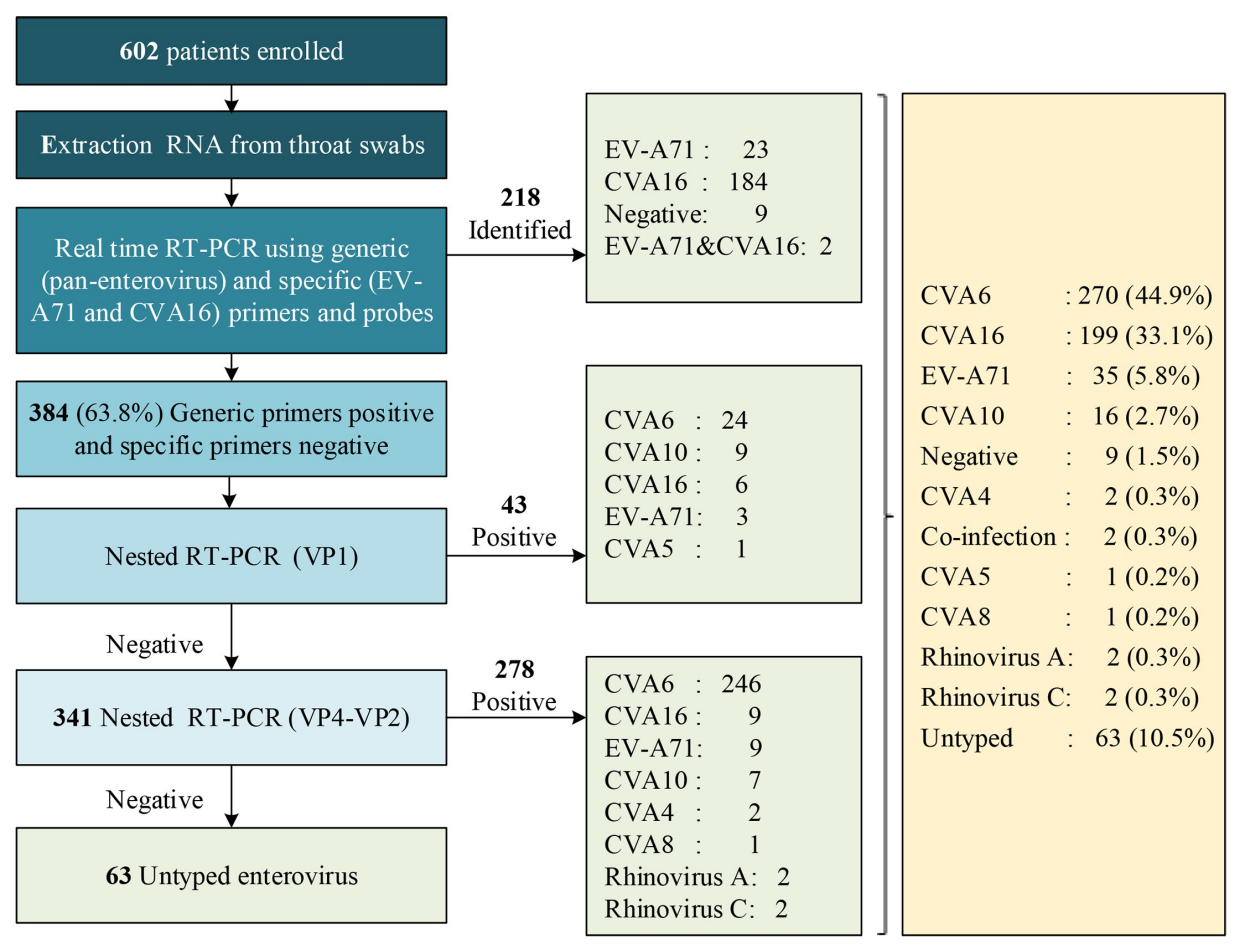
Overview of the testing assay procedures and typing results in Zhengzhou City (Study 2).

An overview of the testing assay procedures used in both studies is described in Figs 1 and 2. In Study 1, stool samples from the patients were collected as soon as possible during their hospitalization, and these samples were the preferred clinical sample for typing. If a specific EV serotype was not identified from the stool sample, other sample types, such as rectal and/or throat swabs, were used for further virological diagnosis. In Study 2, only throat swabs were collected for EV typing.

The samples were first extracted using the QIAamp Viral RNA Mini Kit (QIAGEN, Germany) according to the manufacturer’s instructions. A stepwise strategy, combining real-time RT-PCR and several RT-snPCRs, was used for diagnosis. Briefly, in Study 1, EVs were first detected by real-time RT-PCR using generic (pan-enterovirus) and specific (EV-A71, CVA16 and CVA6) primers and probes [23], and RT-snPCRs of the VP1 [22] and VP4 regions [25] were then performed to detect the samples, which yielded positive results in the generic RT-PCRs and negative results in the specific real-time RT-PCRs. If a specific enterovirus serotype remains unidentified, a new primer set was used to amplify the VP4-VP2 region (S1 Table in S1 File) (see Fig 1). In Study 2, EVs were first detected by real-time RT-PCR using generic (pan-enterovirus) and specific (EV-A71 and CVA16) primers and probes, and RT-snPCRs of the VP1 and VP4-VP2 regions were then performed to detect the samples, which yielded positive results in the generic RT-PCRs and negative results in the specific real-time RT-PCRs (see Fig 2).

Based on the VP4-VP2 region, RT-snPCR was performed as follows: the first round of amplification was performed with the primer mix 458-F, HEVA_VP4_1217a, HEVB_VP4_1215a, and HEVC_VP4_1214a (working concentration: 10 uM), in a volume of 50 μl volume using the first-round primers and the SuperScript III One-Step RT-PCR kit (Invitrogen). The cycling parameters for the first-round of RT-PCR were as follows: an initial incubation at 42°C for 60 min, an initial denaturation at 95°C for 3 min, 25 cycles of denaturation at 94°C for 30 s, annealing at 50°C for 30 s, and extension at 72°C for 30 s and a final extension at 72°C for 10 min.

The second-round of PCR amplification was performed with the primer mix 547-F, HEVA_VP4_1178a, HEVB_VP4_1178a, and HEVC_VP4_1178a in a volume of 25 μl using DreamTaq Green PCR Master Mix (Invitrogen). The cycling parameters were the same as those described above. The expected size of the PCR products was approximately 630 bp and EV-A71 and CVA16 isolates were used as positive controls in all the experiments.

All PCR products were sequenced using the Big Dye Terminator v3.1 Cycle Sequencing Kit (Applied Biosystems, USA) and the PCR primers in both the forward and reverse directions with a 96-capillary 3730xl DNA Analyzer (Applied Biosystems, USA).

The sequences were identified by BLAST (https://blast.ncbi.nlm.nih.gov/Blast.cgi).

The typing results were confirmed by retesting, which was performed by Shanghai BioGerm Medical Biotechnology Co., Ltd. A random subset of samples was selected from each serotype (CVA16, CVA6 and EV-A71) and comprised 10% of all clinical specimens from both studies.

### Data analysis

To confirm the sample viral loads, the Ct values obtained for the typed and untyped samples were compared using Student’s t-test. The data were cleaned using Microsoft Excel 2010, and the analyses were conducted using SPSS version 19.0.

### Ethical approval

Study 1 was approved by the ethical review committees at the Chinese Center for Disease Control and Prevention and the World Health Organization Regional Office for the Western Pacific [23]. Study 2 was approved by the ethical review committees at the Chinese Center for Disease Control and Prevention, the Public Health School of Fudan University and Henan Children’s Hospital [24]. Verbal informed consent was obtained from all the patients’ parents or guardians.

## Results

### Overview of typing results

An overview of the testing procedure, detection process and typing results are shown in Figs 1 and 2. In Study 1 (Anhua County), 2,836 patients were enrolled, 2,517 of these patients (88.8%) were diagnosed with EV infection, and only four of these infections (<0.5%) could not be typed. The most frequently detected serotypes were CVA16 (28.7%, 819), CVA6 (27.7%, 785) and EV-A71 (18.1%, 513). In Study 2 (Zhengzhou city), 602 patients were enrolled, 593 patients (89.5%) were diagnosed with EV infection, and 63 of these patients (10.5%) remained untyped. The most frequently detected serotypes were CVA6 (44.9%, 270), CVA16 (33.1%, 199) and EV-A71 (5.8%, 35) (S2 Table in S1 File).

The combination of the results from Studies 1 and 2 revealed that the dominant serotype in 2013, 2015 and 2018 was CVA6. Moreover, the dominant serotype in 2014 was CVA16, the dominant serotypes in 2016 were CVA16 and EV-A71, and those in 2017 were CVA16 and CVA6 (Table 1).

**Table 1:** Distribution of different EVs in different years.

****Serotype****	****Year, n (%)****					
	****2013****	****2014****	****2015****	****2016****	****2017****	****2018****
****CVA16****	16 (9.1)	580 (49.3)	24 (4.9)	199 (29.6)	182 (40.3)	17 (12.1)
****EV-A71****	8 (4.6)	216 (18.4)	84 (17.0)	205 (30.5)	33 (7.3)	2 (1.4)
****CVA6****	137 (78.3)	194 (16.5)	305 (61.7)	149 (22.2)	167 (36.9)	103 (73.0)
****Others****	14 (8.0)	186 (15.8)	81 (16.4)	119 (17.7)	70 (15.5)	19 (13.5)

Note: The data from 2013–2016 were obtained in Study 1, and the data from 2017–2018 were obtained in Study 2.

### Serotyping methodology strategy for HFMD clinical specimens

In both Studies 1 and 2, 2,107 of 2,836 (74.3%) specimens (EV-A71, CVA16 and CVA6) and 209 of 602 (34.7%) specimens (EV-A71 and CVA16) were found to be positive by real-time RT-PCR. The RT-snPCR assays based on VP1 and VP4-VP2 all showed high sensitivity for downstream typing.

The results showed excellent yield for detection and typing using our serotyping strategy (Study 1: 99.9%; Study 2: 89.5%).

### Investigation of the influence of the Ct value in the typing success

In Study 2, low viral loads of the 63 untyped samples might at least partially explain the inability to type the samples that yielded positive results in the general PCR. The results from Study 2 showed that 63% (334/530) of the typed samples had a Ct value lower than 30, whereas only 30% (19/63) among the untyped specimens had a Ct value lower than 30 (S3 Table in S1 File). This difference was supported by further investigation, which demonstrated significant (p-value < 0.001, Student’s t-test) differences in the Ct values between the typed and untyped samples tested by RT-snPCR (for further details see S1 File). Sample Ct values higher than 30 serves as an explanatory factor of the viral typing failure.

### Investigation of the variation among primer binding sites in real-time RT-PCR

The results of our study showed that CVA16 (Study 1: 15, 1.8%; Study 2: 15, 7.5%), CVA6 (Study 1: 11, 1.4%) and EV-A71 (Study 1: 35, 6.8%; Study 2: 12, 34.2%) were detected in our downstream assays, whereas these were not identified in the specific real-time RT-PCRs. To confirm these results, approximately 10% (n = 347) of all clinical specimens of each serotype (CVA16, CVA6 and EV-A71) collected across both studies were randomly selected for retesting by Shanghai BioGerm Medical Biotechnology Co., Ltd. The external testing of a subset of positive samples indicated 96% typing agreement (n = 333/347) between the testing labs. However, the analyses of the real-time RT-PCR primers and probes binding sites showed that the target amplification sites of EV-A71 downstream primer (10.0%, 2/20), CVA16 downstream primer (5.3%, 1/19), CVA6 upstream primer (5.3%, 1/19), and CVA6 downstream primer (5.3%, 1/19) partly deviated from the designed primer binding site (S1 Fig in S1 File).

### Investigation of the proportion of EVs detection in different clinical specimens

In our previous study, the proportion of EVs detection in the stool samples, rectal swabs and throat swabs in Study 1 were 89% (679/759), 77% (1448/1875) and 74% (99/133), respectively (P < 0.001) [23]. In addition, if stools were tested first and yielded negative results, which resulted in the testing of throat swabs, the proportion of EVs detection increased to 93% (693/746). In contrast, if rectal swabs were tested first and yielded negative results, which resulted in the analysis of throat swabs, the proportion of EVs detection was increased to 89% (1662/1875) [23].

**Fig 3:**
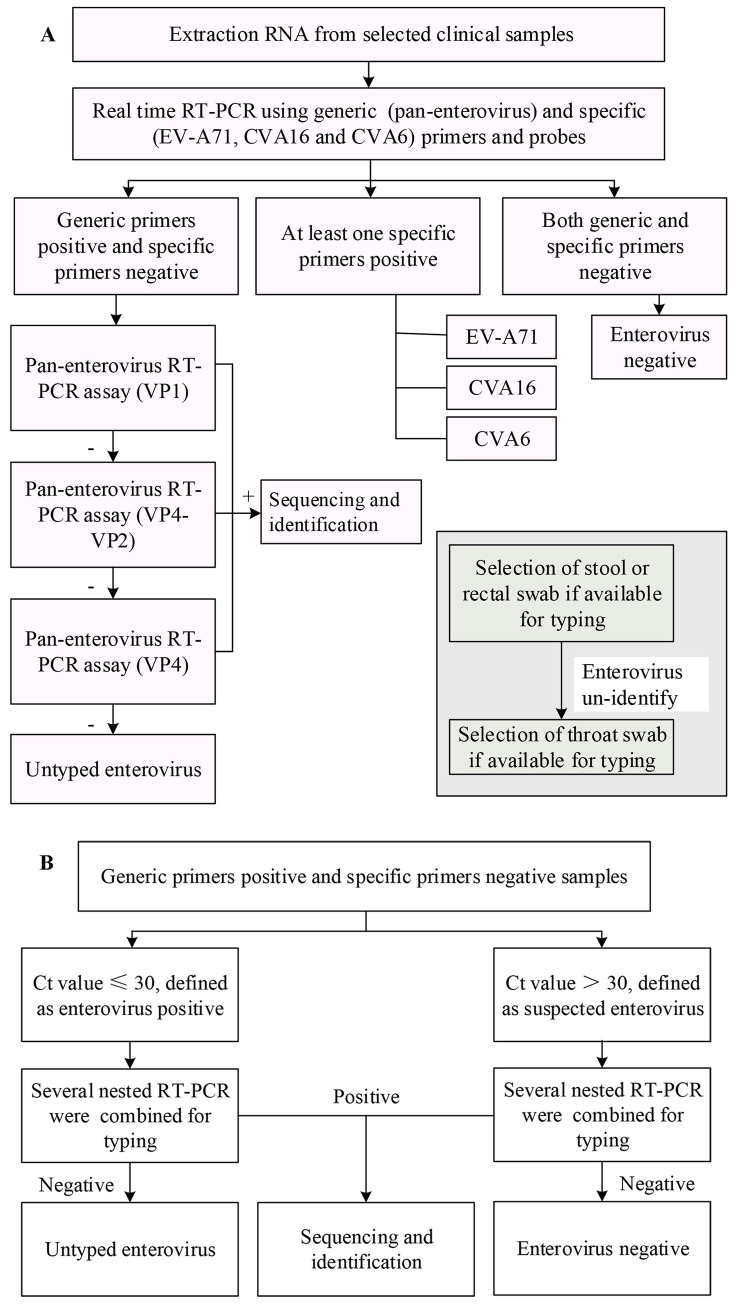
Methodology of the serotyping strategy for HFMD clinical specimens. **3A** –Flowchart depicting the serotyping procedure. **3B –**Flowchart depicting the serotyping procedure for samples identified using the ‘generic primers’ and unbale identified using the ‘specific primers’. Note: “+” indicates an EV-positive result; “-” indicates an EV-negative result.

Here, we propose an efficient molecular strategy for the direct detection and typing of human EVs in clinical specimens associated with HFMD (Fig 3A). A brief description of this strategy is the following: 1) Lab procedure: EVs were first detected by real-time RT-PCR using generic (pan-enterovirus) and specific (EV-A71, CVA16 and CVA6) primers and probes, and RT-snPCRs based sequentially on the VP1, VP4-VP2 and VP4 region s was then performed; 2) Clinical sample selection: The stool sample is selected first, and if a stool sample is not available, the rectal swabs sample is selected; if an EVs failed to be identified, throat swabs from the same patient if available can be used for further diagnosis. Furthermore, if the samples yield positive results in the generic RT-PCR and negative results in the specific RT-PCRs, and the Ct value is higher than 30, the samples can be classified as suspected positive of EVs. If suspected positive samples could not be amplified by RT-snPCRs based on the above-described strategy, the samples were classified as “EVs negative” (Fig 3B). This strategy allows identification of the full spectrum of EVs infections associated with HFMD and will be a valuable tool for clinical virological diagnosis.

## Discussion

National HFMD surveillance in China mainly captures two EVs serotypes: EV-A71 and CVA16 [26]. Over the last decade, the predominant EVs serotypes responsible for HFMD have shifted between epidemic seasons. Previous, HFMD outbreaks were mainly caused by EV-A71 and CVA16, but since 2008, CVA6 and CVA10 have emerged worldwide [27–29]. The EVs typing performed in the two HFMD virological surveillances studies included in the present work, in northern and southern China also detected this trend. During the study period between 2013 and 2018, although CVA16, CVA6 and EV-A71 were the main epidemic strains of EVs, EV-A71 declined in 2017, and the dominant serotype switched between CVA16 and CVA6. CVA6 became the main HFMD causative agent in 2013, 2015, 2017 and 2018, which is consistent with the data reported in 2013, 2015, and 2017, where non-EV-A71 and non-CVA16 EVs dominated the HFMD surveillance system in China [15–17]. The above results indicate that, except for EV-A71 and CVA16 viruses, the CVA6 virus should also be included when formulating a HFMD surveillance system and a strategy for the etiological diagnosis during routine clinical practice.

In this study, we obtained a high diagnostic yield (Study 1: 99.9%; Study 2: 89.5%) with a molecular detection and typing strategy for human EVs in two comprehensive virological surveillance projects of HFMD in northern (Zhengzhou City, Henan Province) and southern (Anhua County, Hunan Province) China. Compared with previous studies, the molecular strategy utilized in this study can directly detect human EVs in clinical specimens associated with HFMD and significantly improves the success proportion of EVs typing. For example, in the study conducted by Ooi MH, et al. [30], the molecular strategy with the highest proportion of EVs typing (78%) consisted of the following: isolated viruses were typed by nucleotide sequencing of the VP1 or VP4 regions, and a combination of clinical samples (throat swabs, vesicle swabs, rectal swabs and ulcer swabs) was used in the diagnosis. In the study conducted by Chen JF, et al. [31], real-time RT-PCR (targeting EV-A71 and CVA16) and several RT-snPCRs (based on the VP1 and VP4 regions) were used in the diagnosis, the clinical samples involved combinations of throat swabs, rectal swabs, and/or stool specimens, and 9.4% (51/545) were unable to be identified. In the study conducted by Gao LD, et al. [23], the molecular strategy involved the following: viral RNA was amplified using generic (pan-enterovirus) and specific (EV-A71, CV-A16, and CV-A6) primers and probes, and if a sample yielded positive results in the generic RT-PCR and negative results in the specific RT-PCRs, a RT-snPCR was used to amplify a portion of the VP1 region [22]. If a specific EV was identified from the stool samples, another type of clinical sample (rectal swab or throat swab) from the same patient was not tested; otherwise, the other sample (if available) was used for further virological diagnosis, but 4% of the samples (116 of 2836) could still not be serotyped.

The results of our study also showed that CVA16, CVA6 and EV-A71 were partially detected in our downstream assays even though they yielded negative results in the specific real-time RT-PCRs. However, further external testing of a subset of positive samples showed 96% typing agreement (n = 333/347) between the testing methods, which indicated the high reliability of our diagnostic strategy. Therefore, the finding might be due to the sensitivity of the assay, but this set of primers was published in 2012 [32] and 2013 [33]. The finding might also be due to the fact that EVs evolve over time, and therefore, the target amplification sites had partly evolved away from the designed primer binding sites.

Additionally, the typing success varied between the two studies, and four samples (0.1%) in Study 1 and 63 samples (10.5%) in Study 2 could not be typed. This variance might be due to differences in the molecular strategy used in the different studies because the samples collected at Zhengzhou City were not examined using the specific CVA6 real-time RT-PCR or the VP4 RT-snPCR. Furthermore, only throat swabs were collected and analyzed in Study 2, whereas in Study 1, if a specific EV was not identified from the primary stool sample, other sample types (rectal swab and throat swab) were collected for further virological diagnosis if possible.

The main limitation of our proposed molecular strategy is observed when all PCR steps fail to detect any EVs in the clinical specimens. Furthermore, the EV-A71 downstream primer had two mutations with subgenogroup C4a isolates, the CVA16 downstream primer had one mutation with subgenogroup B1b isolates, the CVA6 upstream primer and downstream primer respectively had one mutation with subgenogroup D3a isolates in an alignment I performed, which indicated that the primers and probes used in the specific RT-PCRs need to be regularly updated according to the sequences of circulating strains to maintain the effectiveness of the assay. Despite this limitation, our study showed that our strategy exhibited a high diagnostic yield and provided a comprehensive description of the spectrum of EVs serotypes associated with HFMD: only four of 2836 samples (0.1%) in Study 1 and 63 of 593 samples (10.5%) in Study 2 remained untyped.

In conclusion, our findings demonstrate the appropriate application of PCR methods and identify the useful combination of biological sample types for the etiological study of HFMD. Furthermore, this study establishes an effective molecular strategy for the direct detection of all human EVs in clinical specimens associated with HFMD. Additionally, this enhanced virological data will strengthen HFMD surveillance and thus provide a crucial evidentiary basis in the post EV-A71 vaccine era. This strategy will allow an improved evaluation of the impact of the EV-A71 vaccine in China and the continued monitoring of epidemiological and ecological dynamic changes, particularly the potential replacement of EVs serotype.

## Supporting information

S1 File (DOCX)

## Abbreviations

HFMD: Hand, foot, and mouth diseaseHEV-A: human enteroviruses species A
VP: viral proteins
RT: reverse transcription
PCR: Polymerase Chain Reaction
EV: enterovirus
CV: Coxsackievirus

## References

[1] Tapparel C, Siegrist F, Petty TJ, Kaiser L. Picornavirus and enterovirus diversity with associated human diseases. Infection, genetics and evolution: journal of molecular epidemiology and evolutionary genetics in infectious diseases. 2013;14:282–93. Epub 2012/12/04. 10.1016/j.meegid.2012.10.016 . 23201849

[2] B'Krong N, Minh NNQ, Qui PT, Chau TTH, Nghia HDT, Do LAH, et al Enterovirus serotypes in patients with central nervous system and respiratory infections in Viet Nam 1997–2010. Virology journal. 2018;15(1):69 Epub 2018/04/14. 10.1186/s12985-018-0980-0 29650033 PMC5897964

[3] Knipe DM, Howley PM. Fields Virology, 6th Edition: Lippincott Williams & Wilkins; 2013.

[4] Muehlenbachs A, Bhatnagar J, Zaki SR. Tissue tropism, pathology and pathogenesis of enterovirus infection. The Journal of pathology. 2015;235(2):217–28. Epub 2014/09/12. 10.1002/path.4438 . 25211036

[5] Yu H, Cowling BJ. Remaining challenges for prevention and control of hand, foot, and mouth disease. The Lancet Child & adolescent health. 2019;3(6):373–4. Epub 2019/03/20. 10.1016/S2352-4642(19)30065-3 . 30885695

[6] Centers for Disease C, Prevention. Notes from the field: severe hand, foot, and mouth disease associated with coxsackievirus A6—Alabama, Connecticut, California, and Nevada, November 2011-February 2012. MMWR Morbidity and mortality weekly report. 2012;61(12):213–4. . 22456122

[7] Sinclair C, Gaunt E, Simmonds P, Broomfield D, Nwafor N, Wellington L, et al Atypical hand, foot, and mouth disease associated with coxsackievirus A6 infection, Edinburgh, United Kingdom, January to February 2014. Euro surveillance: bulletin Europeen sur les maladies transmissibles = European communicable disease bulletin. 2014;19(12):20745 Epub 2014/04/05. 10.2807/1560-7917.es2014.19.12.20745 . 24698138

[8] Yan X, Zhang ZZ, Yang ZH, Zhu CM, Hu YG, Liu QB. Clinical and Etiological Characteristics of Atypical Hand-Foot-and-Mouth Disease in Children from Chongqing, China: A Retrospective Study. BioMed research international. 2015;2015:802046 10.1155/2015/802046 26693489 PMC4674665

[9] Muppa R, Bhupatiraju P, Duddu M, Dandempally A. Hand, foot and mouth disease. Journal of the Indian Society of Pedodontics and Preventive Dentistry. 2011;29(2):165–7. Epub 2011/09/14. 10.4103/0970-4388.84692 . 21911958

[10] Sapia EY, Maroni C, Groisman C, Kromer H, Lihue Rojo G, Dastugue M, et al Atypical hand-foot-mouth disease virus genotyping in a pediatric hospital in Buenos Aires city, Argentina (in Chinese). Archivos argentinos de pediatria. 2020;118(2):e199–e203. Epub 2020/03/22. 10.5546/aap.2020.e199 . 32199065

[11] Li XW, Ni X, Qian SY, Wang Q, Jiang RM, Xu WB, et al Chinese guidelines for the diagnosis and treatment of hand, foot and mouth disease (2018 edition). World journal of pediatrics: WJP. 2018;14(5):437–47. 10.1007/s12519-018-0189-8 . 30280313

[12] Solomon T, Lewthwaite P, Perera D, Cardosa MJ, McMinn P, Ooi MH. Virology, epidemiology, pathogenesis, and control of enterovirus 71. The Lancet Infectious diseases. 2010;10(11):778–90. Epub 2010/10/22. 10.1016/S1473-3099(10)70194-8 . 20961813

[13] Li Y, Chang Z, Wu P, Liao Q, Liu F, Zheng Y, et al Emerging Enteroviruses Causing Hand, Foot and Mouth Disease, China, 2010–2016. Emerging infectious diseases. 2018;24(10):1902–6. Epub 2018/09/19. 10.3201/eid2410.171953 30226172 PMC6154135

[14] http://www.nhc.gov.cn/jkj/. Accessed: June 2 2020.

[15] Yang B, Liu F, Liao Q, Wu P, Chang Z, Huang J, et al Epidemiology of hand, foot and mouth disease in China, 2008 to 2015 prior to the introduction of EV-A71 vaccine. Euro surveillance: bulletin Europeen sur les maladies transmissibles = European communicable disease bulletin. 2017;22(50):16–00824. Epub 2017/12/21. 10.2807/1560-7917.ES.2017.22.50.16-00824 29258646 PMC5743100

[16] Li JL, Yuan J, Yang F, Wu ZQ, Hu YF, Xue Y, et al Epidemic characteristics of hand, foot, and mouth disease in southern China, 2013: coxsackievirus A6 has emerged as the predominant causative agent. The Journal of infection. 2014;69(3):299–303. Epub 2014/04/16. 10.1016/j.jinf.2014.04.001 . 24731881

[17] Hongyan G, Chengjie M, Qiaozhi Y, Wenhao H, Juan L, Lin P, et al Hand, foot and mouth disease caused by coxsackievirus A6, Beijing, 2013. The Pediatric infectious disease journal. 2014;33(12):1302–3. Epub 2014/07/19. 10.1097/INF.0000000000000467 . 25037037

[18] Harvala H, Broberg E, Benschop K, Berginc N, Ladhani S, Susi P, et al Recommendations for enterovirus diagnostics and characterisation within and beyond Europe. J Clin Virol. 2018;101:11–7. Epub 2018/02/08. 10.1016/j.jcv.2018.01.008 . 29414181

[19] Oberste MS, Maher K, Kilpatrick DR, Pallansch MA. Molecular evolution of the human enteroviruses: correlation of serotype with VP1 sequence and application to picornavirus classification. Journal of virology. 1999;73(3):1941–8. 10.1128/JVI.73.3.1941-1948.1999 9971773 PMC104435

[20] Oberste MS, Maher K, Flemister MR, Marchetti G, Kilpatrick DR, Pallansch MA. Comparison of classic and molecular approaches for the identification of untypeable enteroviruses. Journal of clinical microbiology. 2000;38(3):1170–4. Epub 2000/03/04. 10.1128/JCM.38.3.1170-1174.2000 10699015 PMC86366

[21] Adewumi OM, Faleye TOC, Okeowo CO, Oladapo AM, Oyathelemhi J, Olaniyi OA, et al Identification of previously untypable RD cell line isolates and detection of EV-A71 genotype C1 in a child with AFP in Nigeria. Pathogens and global health. 2018;112(8):421–7. Epub 2018/11/27. 10.1080/20477724.2018.1548117 30474520 PMC6327568

[22] Nix WA, Oberste MS, Pallansch MA. Sensitive, seminested PCR amplification of VP1 sequences for direct identification of all enterovirus serotypes from original clinical specimens. Journal of clinical microbiology. 2006;44(8):2698–704. Epub 2006/08/08. 10.1128/JCM.00542-06 16891480 PMC1594621

[23] Gao L, Zou G, Liao Q, Zhou Y, Liu F, Dai B, et al Spectrum of Enterovirus Serotypes Causing Uncomplicated Hand, Foot, and Mouth Disease and Enteroviral Diagnostic Yield of Different Clinical Samples. Clinical infectious diseases: an official publication of the Infectious Diseases Society of America. 2018;67(11):1729–35. Epub 2018/04/25. 10.1093/cid/ciy341 . 29688329

[24] Cui P, Li Y, Zhou C, Zhou Y, C S. Clinical characteristics and prognosis of mild outpatient hand foot and mouth diseases in children caused by different enteroviruses. Chinese Journal of Pediatrics. 2019;57(6):445–51. 10.3760/cma.j.issn.0578-1310.2019.06.009 31216802

[25] Ishiko H, Shimada Y, Yonaha M, Hashimoto O, Hayashi A, Sakae K, et al Molecular diagnosis of human enteroviruses by phylogeny-based classification by use of the VP4 sequence. The Journal of infectious diseases. 2002;185(6):744–54. Epub 2002/03/29. 10.1086/339298 . 11920292

[26] Tao Z, Wang H, Li Y, Liu G, Xu A, Lin X, et al Molecular epidemiology of human enterovirus associated with aseptic meningitis in Shandong Province, China, 2006–2012. PloS one. 2014;9(2):e89766 Epub 2014/03/04. 10.1371/journal.pone.0089766 24587020 PMC3931826

[27] Blomqvist S, Klemola P, Kaijalainen S, Paananen A, Simonen ML, Vuorinen T, et al Co-circulation of coxsackieviruses A6 and A10 in hand, foot and mouth disease outbreak in Finland. J Clin Virol. 2010;48(1):49–54. 10.1016/j.jcv.2010.02.002 . WOS:000276669700012. 20189452

[28] Song Y, Zhang Y, Ji TJ, Gu XR, Yang Q, Zhu SL, et al Persistent circulation of Coxsackievirus A6 of genotype D3 in mainland of China between 2008 and 2015. Sci Rep-Uk. 2017;7 Artn 5491 10.1038/s41598-017-05618-0 WOS:000405464200128. 28710474 PMC5511160

[29] Wang X, Kwak KJ, Yang Z, Zhang A, Zhang X, Sullivan R, et al Extracellular mRNA detected by molecular beacons in tethered lipoplex nanoparticles for diagnosis of human hepatocellular carcinoma. PloS one. 2018;13(6):e0198552 Epub 2018/06/08. 10.1371/journal.pone.0198552 PubMed Central PMCID: PMC5991670. 29879168 PMC5991670

[30] Ooi MH, Solomon T, Podin Y, Mohan A, Akin W, Yusuf MA, et al Evaluation of different clinical sample types in diagnosis of human enterovirus 71-associated hand-foot-and-mouth disease. Journal of clinical microbiology. 2007;45(6):1858–66. Epub 2007/04/21. 10.1128/JCM.01394-06 17446325 PMC1933032

[31] Chen JF, Zhang RS, Ou XH, Chen FM, Sun BC. The role of enterovirus 71 and coxsackievirus A strains in a large outbreak of hand, foot, and mouth disease in 2012 in Changsha, China. International journal of infectious diseases: IJID: official publication of the International Society for Infectious Diseases. 2014;28:17–25. Epub 2014/09/23. 10.1016/j.ijid.2014.07.024 . 25236389

[32] Zhang L, Wang X, Zhang Y, Gong L, Mao H, Feng C, et al Rapid and sensitive identification of RNA from the emerging pathogen, coxsackievirus A6. Virology journal. 2012;9:298 Epub 2012/12/01. 10.1186/1743-422X-9-298 23194501 PMC3566960

[33] Cui A, Xu C, Tan X, Zhang Y, Zhu Z, Mao N, et al The development and application of the two real-time RT-PCR assays to detect the pathogen of HFMD. PloS one. 2013;8(4):e61451 Epub 2013/05/03. 10.1371/journal.pone.0061451 23637836 PMC3630163

