# Supplementary material for: Molecular strategy for the direct detection and identification of human enteroviruses in clinical specimens associated with hand, foot and mouth disease
Source: PLoS One. 2020 Nov 9;15(11):e0241614. doi: 10.1371/journal.pone.0241614 (PMC7652283; doi:10.1371/journal.pone.0241614)
Supplement: S1 File — (DOCX) [file pone.0241614.s001.docx]

**Molecular strategy for the direct detection and identification of human enteroviruses in clinical specimens associated with hand, foot and mouth disease**

Yonghong Zhou^1^, Qi Qiu^1^, Kaiwei Luo^2^, Qiaohong Liao^1,3^, Yu Li^3^, Peng Cui^1^, Lu Liang^4^, Yibing Cheng^5^, Lili Wang^1^, Kai Wang^1^, Le Van Tan^6^, H. Rogier van Doorn^6,7^, Hongjie Yu^1,*^

^1^School of Public Health, Fudan University, Key Laboratory of Public Health Safety, Ministry of Education, Shanghai, China

^2^Hunan Provincial Center for Disease Control and Prevention, Changsha, Hunan Province, China

^3^Division of Infectious Disease, Key Laboratory of Surveillance and Early-warning on Infectious Disease, Chinese Centre for Disease Control and Prevention, Beijing, China

^4^West China School of Public Health, Sichuan University

^5^Hospital Affiliated to Zhengzhou University, Henan Children’s Hospital, Zhengzhou, China

^6^Oxford University Clinical Research Unit, Ho Chi Minh City, Vietnam

^7^Centre for Tropical Medicine and Global Health, Nuffield Department of Medicine, University of Oxford, Oxford, UK

* Corresponding author

E-mail: yhj@fudan.edu.cn.

**Materials**

**This difference differences in Ct values for typed and untyped samples tested with nested RT-PCR**

Throat swabs were only analysed using nested RT-PCR for typing. 384 samples tested positive in the generic, but negative in the specific RT-PCRs. Of these, 43 samples were positive by VP1 based RT-PCR (Median [95% CI] of Ct value: 25.2 [17.9-34.3]), 278 samples were positive by VP4-VP2 based RT-PCR (Median [95% CI] of Ct value: 28.7 [21.5-35.1]), 63 were negative for both (Median [95% CI] of Ct value: 30.6 [25.3-37.5]), and 321 samples were nested RT-PCR positive (Median [95% CI] of Ct value: 28.3 [20.8-34.9]) (S3 Table and S2 Fig). Results of Ct value analysis by Independent Sample T-Test showed that the Ct values of nested RT-PCR positive samples were lower than negative samples (p-value <0.001).


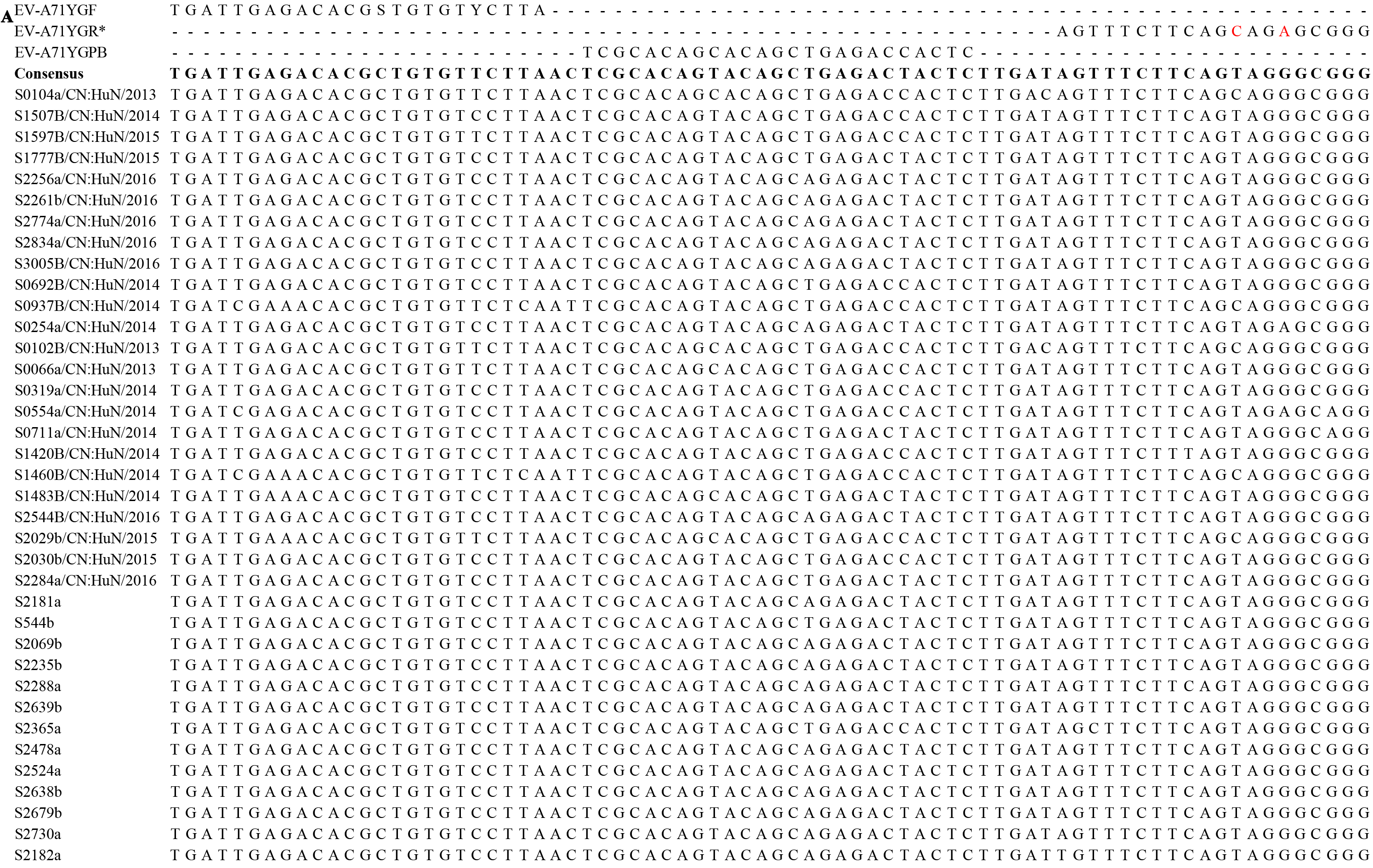


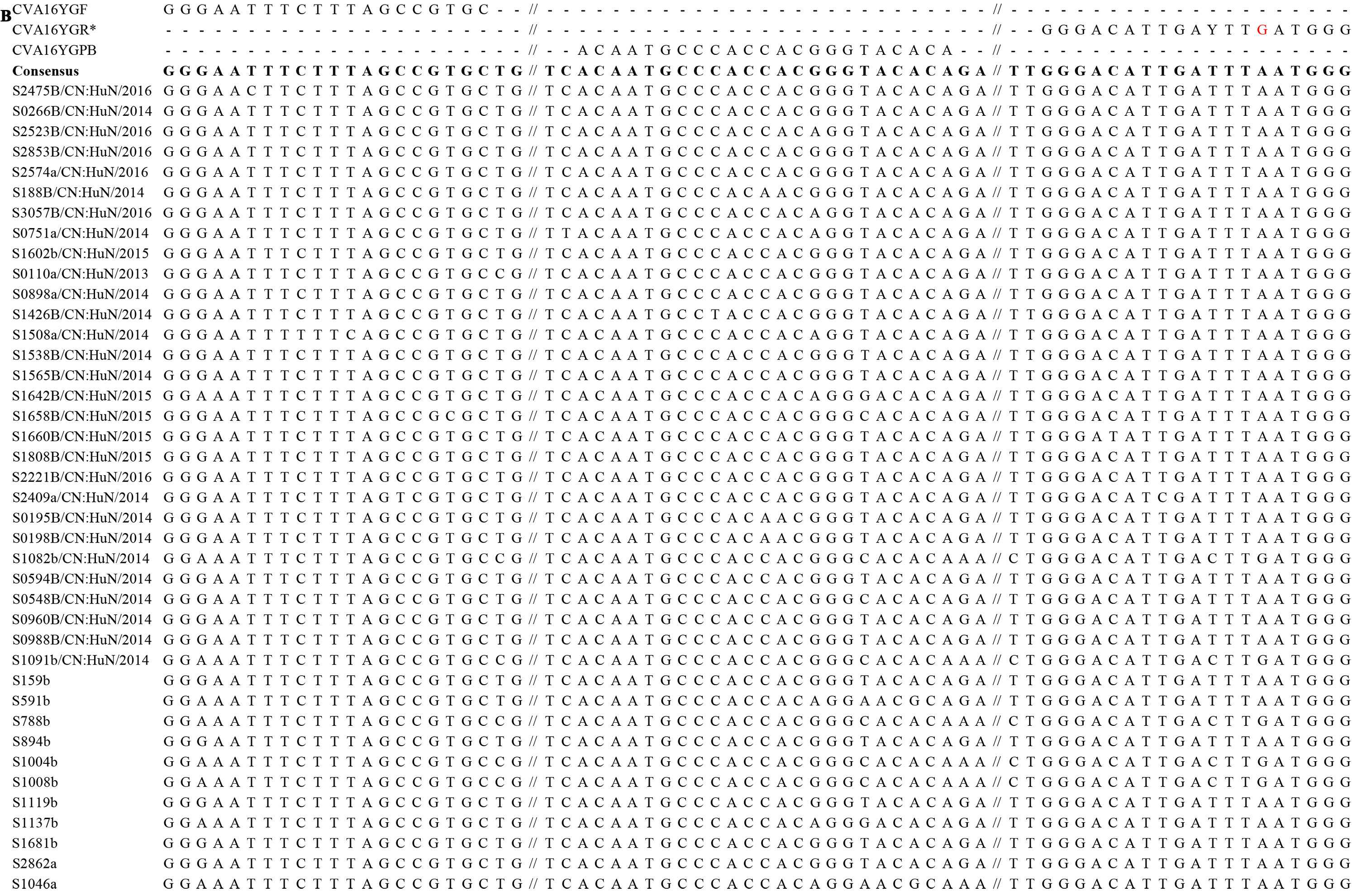


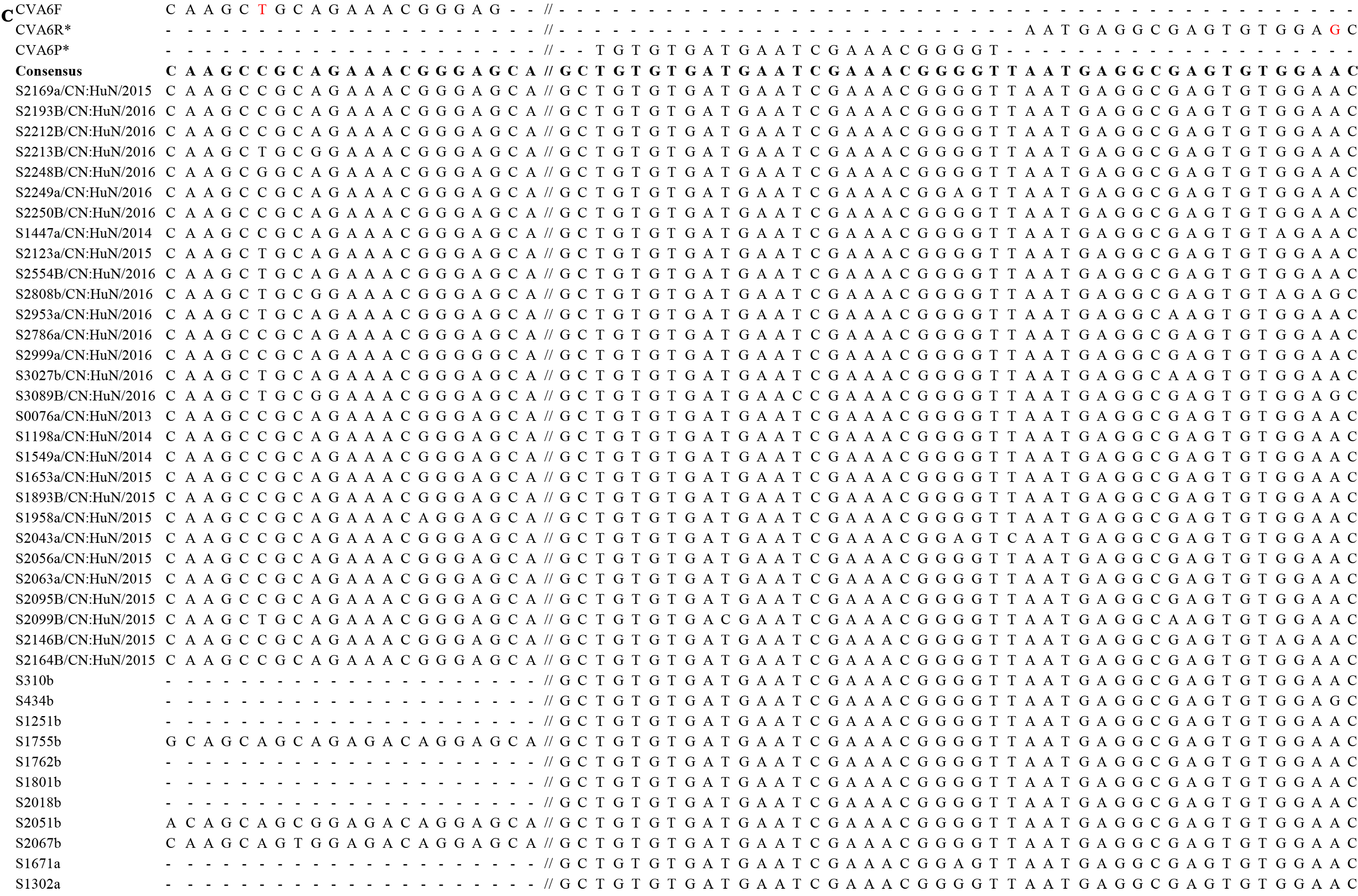


**S1 Fig. Partial changes in the designed primer target amplification sites.** The nucleotide changes in the designed primer target amplification sites are indicated in red.

**A** – EV-A71; **B** – CVA16; **C** – CVA6

The specific primers and probes are aligned with the sequences in our study, and the sequence labeled with CN:HuN indicates that the specific real-time RT-PCRs yielded positive results; the others yielded negative results in the specific real-time RT-PCRs and positive results in the nested RT-PCR based on the VP1 region. For the CVA6 sequences that are not labeled with CN:HuN, “-” indicates that the sequence information in these regions was not obtained, which does not mean the mismatches and gaps in these regions. “*” indicates that the sequence is a reverse complementary sequence.

**
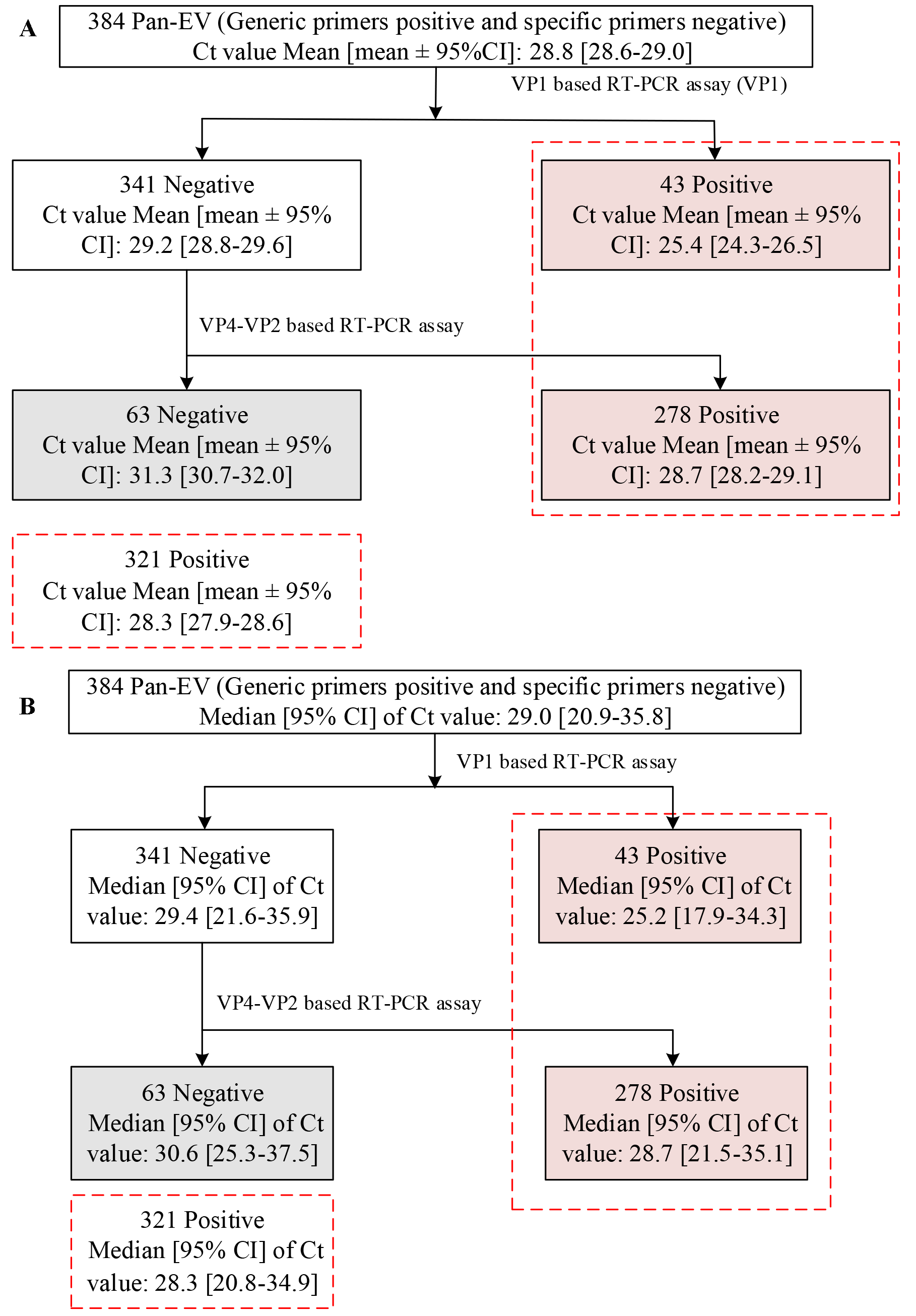
**

**S2 Fig. Ct values for type-able and untype-able independent samples from Study 2 were compared using a Student t test.** **A –** comparison using mean Ct value to analyze different stages of the typing method; **B** **–** comparison using median Ct value to analyze different stages of the typing method.

**S1 Table. The sequences of the primers and probes used in this study**

| **Primer/Probe** | **Sequence (5’🡪3’)** | **Position** | **Gene** | **Methods** |
| --- | --- | --- | --- | --- |
| **EV(YG)F** | GGCTGCGYTGGCGGCC | 361–376 | 5’ UTR | Real-Time RT-PCR |
| **EV(YG)R** | CCAAAGTAGTCGGTTCCGC | 536–554 | 5’ UTR |  |
| **EV (YG)PB** | FAM-CTCCGGCCCCTGAATGCGG-BHQ1 | 450–468 | 5’ UTR |  |
| **EV-A71YGF** | TGATTGAGACACGSTGTGTYCTTA | 2626–2649 | VP1 |  |
| **EV-A71YGR** | CCCGCTCTGCTGAAGAAACT | 2682–2701 | VP1 |  |
| **EV-A71YGPB** | FAM-TCGCACAGCACAGCTGAGACCACTC-BHQ1 | 2652–2676 | VP1 |  |
| **CVA16YGF** | GGGAATTTCTTTAGCCGTGC | 2686–2705 | VP1 |  |
| **CVA16YGR** | CCCATCAARTCAATGTCCC | 2771–2789 | VP1 |  |
| **CVA16YGPB** | FAM-ACAATGCCCACCACGGGTACACA-BHQ1 | 2725–2747 | VP1 |  |
| **CVA6F** | CAAGCTGCAGAAACGGGAG | 2572- 2590 | VP1 |  |
| **CVA6R** | GCTCCACACTCGCCTCATT | 2656- 2674 | VP1 |  |
| **CVA6P** | FAM-ACCCCGTTTCGATTCATCACACA-BHQ1 | 2632-2654 | VP1 |  |
| **SO224** | GCIATGYTIGGIACICAYRT | 1977-1996 | VP3 | Nested RT-PCR (partial VP1) |
| **SO222** | CICCIGGIGGIAYRWACAT | 2969-2951 | VP1 |  |
| **AN32** | GTYTGCCA | 3009-3002 | VP1 |  |
| **AN33** | GAYTGCCA | 3009-3002 | VP1 |  |
| **AN34** | CCRTCRTA | 3111-3104 | VP1 |  |
| **AN35** | RCTYTGCCA | 3009-3002 | VP1 |  |
| **AN89** | CCAGCACTGACAGCAGYNGARAYNGG | 2602-2627 | VP1 |  |
| **AN88** | TACTGGACCACCTGGNGGNAYRWACAT | 2977-2951 | VP1 |  |
| **AN232** | CCAGCACTGACAGCA | 2602-2616 | VP1 |  |
| **AN233** | TACTGGACCACCTGG | 2977-2963 | VP1 |  |
| ***OL68–1** | AYTAAYTTCCACCACCANCC | 1178–1197 | VP2 | RT-PCR (VP4) |
| **MD91** | CCTCCGGCCCCTGAATGCGGCTAAT | 444–468 | VP4 |  |
| **458-F** | CCGGCCCCTGAATGYGGCTAA | 458-478 | VP4 | Nested RT-PCR (VP4-VP2) |
| **HEVA_VP4_1217a** | AYTGNGCRTTYTGNCCRAANACNCC | 1217-1241 | VP2 |  |
| **HEVB_VP4_1215a** | CATRTTYTGNSCRAANARNCCYA | 1215-1237 | VP2 |  |
| **HEVC_VP4_1214a** | ACATRTTYTGNCCRAANADNCCCAT | 1214-1238 | VP2 |  |
| **547-F** | ACCRACTACTTTGGGTGTCCGTG | 547-569 | VP4 |  |
| **HEVA_VP4_1178a** | TCNGGRAAYTTCCARTACCANCC | 1178-1200 | VP2 |  |
| **HEVB_VP4_1178a** | TCNGGNARYTTCCACCACCANCC | 1178-1200 | VP2 |  |
| **HEVC_VP4_1178a** | TCNGGYARYTTCCACCACCANCC | 1178-1200 | VP2 |  |

*OL68-1 were revised according to OL68-1.

**S2 Table. Results of different laboratory testing methods**

| **Method** | **Study 1 (n=2836)** | **Study 2 (n=602)** |
| --- | --- | --- |
| **Real-time RT-PCR (specific RT-PCR positive)** | 2107 (2836, 74.3%) | 209 (602, 34.7%) |
| **Real-time RT-PCR (negative)** | 319 (2836, 11.2%) | 9 (602, 1.5%) |
| **Real-time RT-PCR (generic RT-PCR positive and specific RT-PCR negative)** | 410 (2836, 14.5%) | 384 (602, 63.8%) |
| **Nested RT-PCR (VP1 positive)** | 294 (410, 71.7%) | 43 (384, 11.2%) |
| **RT-PCR (VP4 positive)** | 32 (116, 27.6%) | - |
| **Nested RT-PCR (VP4-VP2 positive)** | 80 (84, 95.2%) | 278 (341, 81.5%) |
| **Total identified** | 2832 (2836, 99.9%) | 539 (602, 89.5%) |
| **Unserotype** | 4 (2836, 0.1%) | 63 (602, 10.5%) |

Note: “-” indicates the experiment was not conducted.

**S3 Table. The testing results in study 2 (Zhengzhou city)**

| **Code** | **Results of real-time RT-PCR** | **Ct value** | **Results of VP1** | **Results of VP4-VP2** | **Results** |
| --- | --- | --- | --- | --- | --- |
| Q1001Y1 | CVA16 | 34.3 | - | - | CVA16 |
| H1002Y1 | CVA16 | 30.75 | - | - | CVA16 |
| Q1004Y1 | CVA16 | 34.31 | - | - | CVA16 |
| H1006Y1 | CVA16 | 29.36 | - | - | CVA16 |
| H1007Y1 | CVA16 | 31.14 | - | - | CVA16 |
| H1011Y1 | CVA16 | 33.78 | - | - | CVA16 |
| H1012Y1 | CVA16 | 34.95 | - | - | CVA16 |
| Q1007Y1 | CVA16 | 32.19 | - | - | CVA16 |
| Q1008Y1 | CVA16 | 28.1 | - | - | CVA16 |
| H1014Y1 | CVA16 | 34.67 | - | - | CVA16 |
| Q1011Y1 | CVA16 | 34.99 | - | - | CVA16 |
| Q1012Y1 | CVA16 | 28.86 | - | - | CVA16 |
| Q1014Y1 | CVA16 | 35.19 | - | - | CVA16 |
| Q1015Y1 | CVA16 | 33.64 | - | - | CVA16 |
| Q1016Y1 | CVA16 | 31.77 | - | - | CVA16 |
| Q1018Y1 | CVA16 | 33.26 | - | - | CVA16 |
| H1020Y1 | CVA16 | 33.29 | - | - | CVA16 |
| H1022Y1 | CVA16 | 30.39 | - | - | CVA16 |
| Q1019Y1 | CVA16 | 33.59 | - | - | CVA16 |
| H1023Y1 | CVA16 | 31.12 | - | - | CVA16 |
| Q1022Y1 | CVA16 | 31.51 | - | - | CVA16 |
| Q1023Y1 | CVA16 | 31.26 | - | - | CVA16 |
| Q1024Y1 | CVA16 | 35.44 | - | - | CVA16 |
| H1026Y1 | CVA16 | 36.25 | - | - | CVA16 |
| H1027Y1 | CVA16 | 36.83 | - | - | CVA16 |
| Q1027Y1 | CVA16 | 32.85 | - | - | CVA16 |
| H1056Y1 | CVA16 | 27.42 | - | - | CVA16 |
| Q1060Y1 | CVA16 | 33.62 | - | - | CVA16 |
| H1054Y1 | CVA16 | 24.29 | - | - | CVA16 |
| Q1050Y1 | CVA16 | 27.86 | - | - | CVA16 |
| Q1049Y1 | CVA16 | 25.66 | - | - | CVA16 |
| H1053Y1 | CVA16 | 23.11 | - | - | CVA16 |
| H1062Y1 | CVA16 | 27.87 | - | - | CVA16 |
| Q1055Y1 | CVA16 | 30.46 | - | - | CVA16 |
| H1059Y1 | CVA16 | 26.27 | - | - | CVA16 |
| H1058Y1 | CVA16 | 31.76 | - | - | CVA16 |
| Q1054Y1 | CVA16 | 28.4 | - | - | CVA16 |
| H1037Y1 | CVA16 | 27.24 | - | - | CVA16 |
| Q1058Y1 | CVA16 | 25.41 | - | - | CVA16 |
| Q1063Y1 | CVA16 | 30.4 | - | - | CVA16 |
| Q1036Y1 | CVA16 | 26.28 | - | - | CVA16 |
| Q1037Y1 | CVA16 | 31.87 | - | - | CVA16 |
| Q1038Y1 | CVA16 | 32.54 | - | - | CVA16 |
| Q1041Y1 | CVA16 | 28.45 | - | - | CVA16 |
| Q1042Y1 | CVA16 | 29.41 | - | - | CVA16 |
| Q1031Y1 | CVA16 | 26.5 | - | - | CVA16 |
| Q1032Y1 | CVA16 | 28.05 | - | - | CVA16 |
| Q1033Y1 | CVA16 | 31.54 | - | - | CVA16 |
| Q1029Y1 | CVA16 | 34.06 | - | - | CVA16 |
| Q1065Y1 | CVA16 | 26.86 | - | - | CVA16 |
| Q1064Y1 | CVA16 | 35.56 | - | - | CVA16 |
| H1070Y1 | CVA16 | 30.83 | - | - | CVA16 |
| H1069Y1 | CVA16 | 28.42 | - | - | CVA16 |
| H1075Y1 | CVA16 | 26.02 | - | - | CVA16 |
| H1076Y1 | CVA16 | 28.76 | - | - | CVA16 |
| H1077Y1 | CVA16 | 24.47 | - | - | CVA16 |
| H1068Y1 | CVA16 | 26.63 | - | - | CVA16 |
| H1066Y1 | CVA16 | 28.26 | - | - | CVA16 |
| H1032Y1 | CVA16 | 29.49 | - | - | CVA16 |
| H1064Y1 | CVA16 | 34.83 | - | - | CVA16 |
| H1063Y1 | CVA16 | 31.85 | - | - | CVA16 |
| H1042Y1 | CVA16 | 25.11 | - | - | CVA16 |
| H1044Y1 | CVA16 | 22.02 | - | - | CVA16 |
| H1047Y1 | CVA16 | 24.44 | - | - | CVA16 |
| H1046Y1 | CVA16 | 24.11 | - | - | CVA16 |
| H1067Y1 | CVA16 | 29.82 | - | - | CVA16 |
| Q1071Y1 | CVA16 | 21.36 | - | - | CVA16 |
| Q1070Y1 | CVA16 | 27.97 | - | - | CVA16 |
| Q1067Y1 | CVA16 | 30.59 | - | - | CVA16 |
| Q1069Y1 | CVA16 | 30.29 | - | - | CVA16 |
| H1074Y1 | CVA16 | 27.19 | - | - | CVA16 |
| H1072Y1 | CVA16 | 27.29 | - | - | CVA16 |
| Q1075Y1 | CVA16 | 26.89 | - | - | CVA16 |
| H1079Y1 | CVA16 | 23.13 | - | - | CVA16 |
| H1080Y1 | CVA16 | 25.75 | - | - | CVA16 |
| H1081Y1 | CVA16 | 25.38 | - | - | CVA16 |
| H1085Y1 | CVA16 | 30.22 | - | - | CVA16 |
| H1086Y1 | CVA16 | 29.67 | - | - | CVA16 |
| Q1078Y1 | CVA16 | 26.7 | - | - | CVA16 |
| Q1077Y1 | CVA16 | 25.51 | - | - | CVA16 |
| H1083Y1 | CVA16 | 25.83 | - | - | CVA16 |
| H1084Y1 | CVA16 | 26.05 | - | - | CVA16 |
| Q1081Y1 | CVA16 | 22.09 | - | - | CVA16 |
| Q1086Y1 | CVA16 | 22.56 | - | - | CVA16 |
| Q1087Y1 | CVA16 | 29.72 | - | - | CVA16 |
| H1090Y1 | CVA16 | 27.16 | - | - | CVA16 |
| Q1090Y1 | CVA16 | 23.26 | - | - | CVA16 |
| Q1089Y1 | CVA16 | 25.16 | - | - | CVA16 |
| H1094Y1 | CVA16 | 29.23 | - | - | CVA16 |
| H1095Y1 | CVA16 | 20.95 | - | - | CVA16 |
| Q1093Y1 | CVA16 | 29.28 | - | - | CVA16 |
| Q1095Y1 | CVA16 | 28.25 | - | - | CVA16 |
| Q1096Y1 | CVA16 | 27.66 | - | - | CVA16 |
| Q1097Y1 | CVA16 | 28.34 | - | - | CVA16 |
| H1097Y1 | CVA16 | 27.2 | - | - | CVA16 |
| H1105Y1 | CVA16 | 24.19 | - | - | CVA16 |
| H1098Y1 | CVA16 | 22.66 | - | - | CVA16 |
| H1106Y1 | CVA16 | 24.49 | - | - | CVA16 |
| H1107Y1 | CVA16 | 29.66 | - | - | CVA16 |
| H1108Y1 | CVA16 | 33.63 | - | - | CVA16 |
| H1111Y1 | CVA16 | 22.76 | - | - | CVA16 |
| H1112Y1 | CVA16 | 29.32 | - | - | CVA16 |
| Q1106Y1 | CVA16 | 24.54 | - | - | CVA16 |
| Q1107Y1 | CVA16 | 26.96 | - | - | CVA16 |
| Q1108Y1 | CVA16 | 28.45 | - | - | CVA16 |
| H1113Y1 | CVA16 | 24.41 | - | - | CVA16 |
| H1114Y1 | CVA16 | 27.35 | - | - | CVA16 |
| H1117Y1 | CVA16 | 28.95 | - | - | CVA16 |
| Q1112Y1 | CVA16 | 26.62 | - | - | CVA16 |
| Q1116Y1 | CVA16 | 32.94 | - | - | CVA16 |
| Q1117Y1 | CVA16 | 31.11 | - | - | CVA16 |
| H1119Y1 | CVA16 | 34.2 | - | - | CVA16 |
| H1120Y1 | CVA16 | 28.95 | - | - | CVA16 |
| H1123Y1 | CVA16 | 26.71 | - | - | CVA16 |
| H1128Y1 | CVA16 | 27.71 | - | - | CVA16 |
| Q1119Y1 | CVA16 | 24.39 | - | - | CVA16 |
| Q1120Y1 | CVA16 | 32.33 | - | - | CVA16 |
| H1131Y1 | CVA16 | 27.23 | - | - | CVA16 |
| H1133Y1 | CVA16 | 27.36 | - | - | CVA16 |
| Q1124Y1 | CVA16 | 36.96 | - | - | CVA16 |
| H1139Y1 | CVA16 | 31.18 | - | - | CVA16 |
| H1140Y1 | CVA16 | 33.4 | - | - | CVA16 |
| H1141Y1 | CVA16 | 34.3 | - | - | CVA16 |
| Q1132Y1 | CVA16 | 28.96 | - | - | CVA16 |
| H1146Y1 | CVA16 | 29.75 | - | - | CVA16 |
| H1147Y1 | CVA16 | 31.5 | - | - | CVA16 |
| H1148Y1 | CVA16 | 29.04 | - | - | CVA16 |
| Q1138Y1 | CVA16 | 30.71 | - | - | CVA16 |
| Q1136Y1 | CVA16 | 31.19 | - | - | CVA16 |
| Q1134Y1 | CVA16 | 30.53 | - | - | CVA16 |
| Q1135Y1 | CVA16 | 29.99 | - | - | CVA16 |
| H1150Y1 | CVA16 | 27.91 | - | - | CVA16 |
| H1149Y1 | CVA16 | 33.33 | - | - | CVA16 |
| Q1140Y1 | CVA16 | 29.88 | - | - | CVA16 |
| Q1142Y1 | CVA16 | 35.47 | - | - | CVA16 |
| Q1145Y1 | CVA16 | 34.2 | - | - | CVA16 |
| H1165Y1 | CVA16 | 29.19 | - | - | CVA16 |
| H1175Y1 | CVA16 | 25.03 | - | - | CVA16 |
| Q1154Y1 | CVA16 | 28.12 | - | - | CVA16 |
| Q1156Y1 | CVA16 | 32.94 | - | - | CVA16 |
| H1187Y1 | CVA16 | 33.43 | - | - | CVA16 |
| H1189Y1 | CVA16 | 36.9 | - | - | CVA16 |
| H1192Y1 | CVA16 | 33.77 | - | - | CVA16 |
| Q1163Y1 | CVA16 | 32.59 | - | - | CVA16 |
| Q1164Y1 | CVA16 | 28.81 | - | - | CVA16 |
| Q1168Y1 | CVA16 | 34.85 | - | - | CVA16 |
| Q1170Y1 | CVA16 | 25.58 | - | - | CVA16 |
| Q1172Y1 | CVA16 | 30.24 | - | - | CVA16 |
| Q1173Y1 | CVA16 | 35.4 | - | - | CVA16 |
| Q1177Y1 | CVA16 | 24.57 | - | - | CVA16 |
| Q1180Y1 | CVA16 | 27.66 | - | - | CVA16 |
| Q1188Y1 | CVA16 | 29.49 | - | - | CVA16 |
| H1198Y1 | CVA16 | 26.39 | - | - | CVA16 |
| Q1198Y1 | CVA16 | 26.49 | - | - | CVA16 |
| Q1199Y1 | CVA16 | 32.58 | - | - | CVA16 |
| Q1213Y1 | CVA16 | 26.92 | - | - | CVA16 |
| Q1226Y1 | CVA16 | 27.41 | - | - | CVA16 |
| Q1232Y1 | CVA16 | 30.05 | - | - | CVA16 |
| Q1240Y1 | CVA16 | 33.65 | - | - | CVA16 |
| Q1241Y1 | CVA16 | 26.66 | - | - | CVA16 |
| Q1244Y1 | CVA16 | 28.27 | - | - | CVA16 |
| Q1247Y1 | CVA16 | 31.83 | - | - | CVA16 |
| Q1253Y1 | CVA16 | 25.12 | - | - | CVA16 |
| H1252Y1 | CVA16 | 31.18 | - | - | CVA16 |
| H1264Y1 | CVA16 | 30.47 | - | - | CVA16 |
| H1286Y1 | CVA16 | 30.8 | - | - | CVA16 |
| H1294Y1 | CVA16 | 32.28 | - | - | CVA16 |
| H1299Y1 | CVA16 | 29.15 | - | - | CVA16 |
| H1301Y1 | CVA16 | 30.48 | - | - | CVA16 |
| H1303Y1 | CVA16 | 28.19 | - | - | CVA16 |
| H1314Y1 | CVA16 | 23.16 | - | - | CVA16 |
| H1309Y1 | CVA16 | 24.83 | - | - | CVA16 |
| H1319Y1 | CVA16 | 29.18 | - | - | CVA16 |
| Q1151Y1 | CVA16 | 32.16 | - | - | CVA16 |
| Q1152Y1 | CVA16 | 32.83 | - | - | CVA16 |
| Q1153Y1 | CVA16 | 35.58 | - | - | CVA16 |
| H1321Y1 | CVA16 | 28.22 | - | - | CVA16 |
| H1158Y1 | CVA16 | 31.23 | - | - | CVA16 |
| H1333Y1 | CVA16 | 33.79 | - | - | CVA16 |
| H1335Y1 | CVA16 | 27.59 | - | - | CVA16 |
| H1337Y1 | CVA16 | 35.66 | - | - | CVA16 |
| H1343Y1 | CVA16 | 29.65 | - | - | CVA16 |
| H1346Y1 | CVA16 | 31.4 | - | - | CVA16 |
| H1348Y1 | CVA16 | 22.45 | - | - | CVA16 |
| H1010Y1 | EV-A71 | 28.77 | - | - | EV-A71 |
| H1016Y1 | EV-A71 | 29.06 | - | - | EV-A71 |
| H1029Y1 | EV-A71 | 32.27 | - | - | EV-A71 |
| Q1051Y1 | EV-A71 | 29.11 | - | - | EV-A71 |
| Q1057Y1 | EV-A71 | 27.59 | - | - | EV-A71 |
| H1036Y1 | EV-A71 | 33.12 | - | - | EV-A71 |
| Q1061Y1 | EV-A71 | 32.12 | - | - | EV-A71 |
| Q1035Y1 | EV-A71 | 28.87 | - | - | EV-A71 |
| Q1046Y1 | EV-A71 | 32.15 | - | - | EV-A71 |
| H1039Y1 | EV-A71 | 30.8 | - | - | EV-A71 |
| Q1068Y1 | EV-A71 | 35.92 | - | - | EV-A71 |
| H1048Y1 | EV-A71 | 23.33 | - | - | EV-A71 |
| H1045Y1 | EV-A71 | 25.87 | - | - | EV-A71 |
| H1089Y1 | EV-A71 | 29.2 | - | - | EV-A71 |
| H1103Y1 | EV-A71 | 28.92 | - | - | EV-A71 |
| H1115Y1 | EV-A71 | 31.07 | - | - | EV-A71 |
| Q1111Y1 | EV-A71 | 37.45 | - | - | EV-A71 |
| H1121Y1 | EV-A71 | 28.28 | - | - | EV-A71 |
| H1129Y1 | EV-A71 | 23.19 | - | - | EV-A71 |
| Q1147Y1 | EV-A71 | 37.86 | - | - | EV-A71 |
| Q1157Y1 | EV-A71 | 35.58 | - | - | EV-A71 |
| H1172Y1 | EV-A71 | 37.86 | - | - | EV-A71 |
| Q1181Y1 | EV-A71 | 31.28 | - | - | EV-A71 |
| Q1066Y1 | EV-A71, CVA16 | 37.61, 27.64 | - | - | EV-A71, CVA16 |
| H1040Y1 | EV-A71, CVA16 | 37.63, 32.69 | - | - | EV-A71, CVA16 |
| Q1059Y1 | Pan-EV | 26.36 | CVA10 | - | CVA10 |
| H1051Y1 | Pan-EV | 17.84 | CVA10 | - | CVA10 |
| Q1052Y1 | Pan-EV | 30.35 | CVA10 | - | CVA10 |
| H1071Y1 | Pan-EV | 23.14 | CVA10 | - | CVA10 |
| H1049Y1 | Pan-EV | 18.48 | CVA10 | - | CVA10 |
| H1104Y1 | Pan-EV | 23.94 | CVA10 | - | CVA10 |
| H1100Y1 | Pan-EV | 24.27 | CVA10 | - | CVA10 |
| Q1109Y1 | Pan-EV | 20.88 | CVA10 | - | CVA10 |
| Q1110Y1 | Pan-EV | 21.67 | CVA10 | - | CVA10 |
| Q1039Y1 | Pan-EV | 34.55 | CVA16 | - | CVA16 |
| H1034Y1 | Pan-EV | 29.06 | CVA16 | - | CVA16 |
| Q1103Y1 | Pan-EV | 32.33 | CVA16 | - | CVA16 |
| H1110Y1 | Pan-EV | 25.22 | CVA16 | - | CVA16 |
| Q1115Y1 | Pan-EV | 28.01 | CVA16 | - | CVA16 |
| H1122Y1 | Pan-EV | 27.11 | CVA16 | - | CVA16 |
| H1118Y1 | Pan-EV | 26.53 | CVA5 | - | CVA5 |
| H1001Y1 | Pan-EV | 26.48 | CVA6 | - | CVA6 |
| H1015Y1 | Pan-EV | 30.75 | CVA6 | - | CVA6 |
| H1024Y1 | Pan-EV | 30.93 | CVA6 | - | CVA6 |
| H1031Y1 | Pan-EV | 27.36 | CVA6 | - | CVA6 |
| Q1026Y1 | Pan-EV | 29.03 | CVA6 | - | CVA6 |
| H1061Y1 | Pan-EV | 20.21 | CVA6 | - | CVA6 |
| Q1043Y1 | Pan-EV | 25.27 | CVA6 | - | CVA6 |
| Q1045Y1 | Pan-EV | 22.65 | CVA6 | - | CVA6 |
| Q1030Y1 | Pan-EV | 30.38 | CVA6 | - | CVA6 |
| H1043Y1 | Pan-EV | 23.76 | CVA6 | - | CVA6 |
| Q1074Y1 | Pan-EV | 23.41 | CVA6 | - | CVA6 |
| H1088Y1 | Pan-EV | 23.48 | CVA6 | - | CVA6 |
| H1091Y1 | Pan-EV | 30.66 | CVA6 | - | CVA6 |
| Q1094Y1 | Pan-EV | 23.05 | CVA6 | - | CVA6 |
| Q1098Y1 | Pan-EV | 28.67 | CVA6 | - | CVA6 |
| H1109Y1 | Pan-EV | 22.23 | CVA6 | - | CVA6 |
| Q1104Y1 | Pan-EV | 26.77 | CVA6 | - | CVA6 |
| H1116Y1 | Pan-EV | 25.65 | CVA6 | - | CVA6 |
| Q1113Y1 | Pan-EV | 24.83 | CVA6 | - | CVA6 |
| Q1114Y1 | Pan-EV | 27.6 | CVA6 | - | CVA6 |
| H1124Y1 | Pan-EV | 21.58 | CVA6 | - | CVA6 |
| H1125Y1 | Pan-EV | 20.79 | CVA6 | - | CVA6 |
| H1130Y1 | Pan-EV | 25.83 | CVA6 | - | CVA6 |
| H1132Y1 | Pan-EV | 23.99 | CVA6 | - | CVA6 |
| Q1082Y1 | Pan-EV | 24.47 | EV-A71 | - | EV-A71 |
| H1093Y1 | Pan-EV | 20.91 | EV-A71 | - | EV-A71 |
| H1092Y1 | Pan-EV | 21.76 | EV-A71 | - | EV-A71 |
| Q1100Y1 | Pan-EV | 25.07 | Negative | CVA10 | CVA10 |
| H1194Y1 | Pan-EV | 32.9 | Negative | CVA10 | CVA10 |
| Q1217Y1 | Pan-EV | 28.09 | Negative | CVA10 | CVA10 |
| Q1250Y1 | Pan-EV | 24.21 | Negative | CVA10 | CVA10 |
| H1230Y1 | Pan-EV | 27.35 | Negative | CVA10 | CVA10 |
| H1323Y1 | Pan-EV | 21.13 | Negative | CVA10 | CVA10 |
| H1332Y1 | Pan-EV | 20.52 | Negative | CVA10 | CVA10 |
| H1052Y1 | Pan-EV | 33.57 | Negative | CVA16 | CVA16 |
| Q1062Y1 | Pan-EV | 34.31 | Negative | CVA16 | CVA16 |
| Q1053Y1 | Pan-EV | 30.64 | Negative | CVA16 | CVA16 |
| Q1044Y1 | Pan-EV | 31.29 | Negative | CVA16 | CVA16 |
| Q1092Y1 | Pan-EV | 29.26 | Negative | CVA16 | CVA16 |
| H1050Y1 | Pan-EV | 27.91 | Negative | CVA16 | CVA16 |
| H1137Y1 | Pan-EV | 27.47 | Negative | CVA16 | CVA16 |
| Q1144Y1 | Pan-EV | 25.44 | Negative | CVA16 | CVA16 |
| Q1150Y1 | Pan-EV | 33.21 | Negative | CVA16 | CVA16 |
| H1003Y1 | Pan-EV | 27.84 | Negative | CVA4 | CVA4 |
| Q1083Y1 | Pan-EV | 31.55 | Negative | CVA4 | CVA4 |
| H1005Y1 | Pan-EV | 31.3 | Negative | CVA6 | CVA6 |
| H1008Y1 | Pan-EV | 32.51 | Negative | CVA6 | CVA6 |
| Q1005Y1 | Pan-EV | 27.43 | Negative | CVA6 | CVA6 |
| Q1009Y1 | Pan-EV | 28.72 | Negative | CVA6 | CVA6 |
| H1013Y1 | Pan-EV | 30.28 | Negative | CVA6 | CVA6 |
| H1018Y1 | Pan-EV | 30.88 | Negative | CVA6 | CVA6 |
| H1019Y1 | Pan-EV | 34.95 | Negative | CVA6 | CVA6 |
| Q1017Y1 | Pan-EV | 34.11 | Negative | CVA6 | CVA6 |
| H1021Y1 | Pan-EV | 29.36 | Negative | CVA6 | CVA6 |
| H1055Y1 | Pan-EV | 25.36 | Negative | CVA6 | CVA6 |
| H1035Y1 | Pan-EV | 26.04 | Negative | CVA6 | CVA6 |
| H1060Y1 | Pan-EV | 28.84 | Negative | CVA6 | CVA6 |
| Q1056Y1 | Pan-EV | 24.1 | Negative | CVA6 | CVA6 |
| H1057Y1 | Pan-EV | 26.88 | Negative | CVA6 | CVA6 |
| Q1040Y1 | Pan-EV | 28.46 | Negative | CVA6 | CVA6 |
| Q1047Y1 | Pan-EV | 27.91 | Negative | CVA6 | CVA6 |
| H1033Y1 | Pan-EV | 33.77 | Negative | CVA6 | CVA6 |
| H1065Y1 | Pan-EV | 26.23 | Negative | CVA6 | CVA6 |
| Q1072Y1 | Pan-EV | 24.27 | Negative | CVA6 | CVA6 |
| Q1073Y1 | Pan-EV | 30.04 | Negative | CVA6 | CVA6 |
| H1078Y1 | Pan-EV | 28.45 | Negative | CVA6 | CVA6 |
| H1087Y1 | Pan-EV | 23.74 | Negative | CVA6 | CVA6 |
| Q1076Y1 | Pan-EV | 25.63 | Negative | CVA6 | CVA6 |
| H1082Y1 | Pan-EV | 30.83 | Negative | CVA6 | CVA6 |
| Q1088Y1 | Pan-EV | 28.78 | Negative | CVA6 | CVA6 |
| Q1099Y1 | Pan-EV | 29.42 | Negative | CVA6 | CVA6 |
| Q1101Y1 | Pan-EV | 28.92 | Negative | CVA6 | CVA6 |
| H1099Y1 | Pan-EV | 24.58 | Negative | CVA6 | CVA6 |
| H1101Y1 | Pan-EV | 28.72 | Negative | CVA6 | CVA6 |
| Q1105Y1 | Pan-EV | 31.37 | Negative | CVA6 | CVA6 |
| H1126Y1 | Pan-EV | 25.27 | Negative | CVA6 | CVA6 |
| H1127Y1 | Pan-EV | 24.17 | Negative | CVA6 | CVA6 |
| Q1122Y1 | Pan-EV | 29.59 | Negative | CVA6 | CVA6 |
| Q1123Y1 | Pan-EV | 27.92 | Negative | CVA6 | CVA6 |
| H1134Y1 | Pan-EV | 27.98 | Negative | CVA6 | CVA6 |
| H1135Y1 | Pan-EV | 27.97 | Negative | CVA6 | CVA6 |
| H1136Y1 | Pan-EV | 28.46 | Negative | CVA6 | CVA6 |
| Q1125Y1 | Pan-EV | 30.88 | Negative | CVA6 | CVA6 |
| Q1126Y1 | Pan-EV | 28.54 | Negative | CVA6 | CVA6 |
| H1138Y1 | Pan-EV | 26 | Negative | CVA6 | CVA6 |
| H1143Y1 | Pan-EV | 25.81 | Negative | CVA6 | CVA6 |
| H1144Y1 | Pan-EV | 24.95 | Negative | CVA6 | CVA6 |
| H1145Y1 | Pan-EV | 25.83 | Negative | CVA6 | CVA6 |
| Q1127Y1 | Pan-EV | 26.3 | Negative | CVA6 | CVA6 |
| Q1128Y1 | Pan-EV | 21.5 | Negative | CVA6 | CVA6 |
| Q1131Y1 | Pan-EV | 26.89 | Negative | CVA6 | CVA6 |
| Q1137Y1 | Pan-EV | 27.71 | Negative | CVA6 | CVA6 |
| Q1133Y1 | Pan-EV | 25.27 | Negative | CVA6 | CVA6 |
| H1151Y1 | Pan-EV | 26.19 | Negative | CVA6 | CVA6 |
| Q1139Y1 | Pan-EV | 25.3 | Negative | CVA6 | CVA6 |
| Q1141Y1 | Pan-EV | 29.66 | Negative | CVA6 | CVA6 |
| H1153Y1 | Pan-EV | 22.07 | Negative | CVA6 | CVA6 |
| Q1143Y1 | Pan-EV | 32.03 | Negative | CVA6 | CVA6 |
| H1157Y1 | Pan-EV | 27.13 | Negative | CVA6 | CVA6 |
| H1156Y1 | Pan-EV | 24.83 | Negative | CVA6 | CVA6 |
| H1155Y1 | Pan-EV | 34.22 | Negative | CVA6 | CVA6 |
| Q1146Y1 | Pan-EV | 25.95 | Negative | CVA6 | CVA6 |
| Q1158Y1 | Pan-EV | 31.6 | Negative | CVA6 | CVA6 |
| Q1159Y1 | Pan-EV | 30.07 | Negative | CVA6 | CVA6 |
| H1168Y1 | Pan-EV | 29.33 | Negative | CVA6 | CVA6 |
| H1169Y1 | Pan-EV | 34.86 | Negative | CVA6 | CVA6 |
| H1162Y1 | Pan-EV | 27.28 | Negative | CVA6 | CVA6 |
| H1163Y1 | Pan-EV | 27.45 | Negative | CVA6 | CVA6 |
| H1170Y1 | Pan-EV | 30.96 | Negative | CVA6 | CVA6 |
| H1166Y1 | Pan-EV | 25.76 | Negative | CVA6 | CVA6 |
| H1164Y1 | Pan-EV | 28.14 | Negative | CVA6 | CVA6 |
| H1173Y1 | Pan-EV | 34.12 | Negative | CVA6 | CVA6 |
| H1174Y1 | Pan-EV | 29.21 | Negative | CVA6 | CVA6 |
| Q1155Y1 | Pan-EV | 30.35 | Negative | CVA6 | CVA6 |
| H1176Y1 | Pan-EV | 32.24 | Negative | CVA6 | CVA6 |
| H1177Y1 | Pan-EV | 31.71 | Negative | CVA6 | CVA6 |
| H1178Y1 | Pan-EV | 26.23 | Negative | CVA6 | CVA6 |
| H1183Y1 | Pan-EV | 25.57 | Negative | CVA6 | CVA6 |
| H1181Y1 | Pan-EV | 27.5 | Negative | CVA6 | CVA6 |
| H1186Y1 | Pan-EV | 28.55 | Negative | CVA6 | CVA6 |
| H1190Y1 | Pan-EV | 31.27 | Negative | CVA6 | CVA6 |
| H1191Y1 | Pan-EV | 29.92 | Negative | CVA6 | CVA6 |
| H1193Y1 | Pan-EV | 28.86 | Negative | CVA6 | CVA6 |
| Q1161Y1 | Pan-EV | 23.59 | Negative | CVA6 | CVA6 |
| Q1162Y1 | Pan-EV | 29.94 | Negative | CVA6 | CVA6 |
| Q1166Y1 | Pan-EV | 29.37 | Negative | CVA6 | CVA6 |
| Q1167Y1 | Pan-EV | 28.14 | Negative | CVA6 | CVA6 |
| Q1169Y1 | Pan-EV | 28.07 | Negative | CVA6 | CVA6 |
| Q1184Y1 | Pan-EV | 27.24 | Negative | CVA6 | CVA6 |
| Q1185Y1 | Pan-EV | 30.78 | Negative | CVA6 | CVA6 |
| Q1186Y1 | Pan-EV | 19.42 | Negative | CVA6 | CVA6 |
| Q1174Y1 | Pan-EV | 34.65 | Negative | CVA6 | CVA6 |
| Q1176Y1 | Pan-EV | 32.08 | Negative | CVA6 | CVA6 |
| Q1178Y1 | Pan-EV | 25.71 | Negative | CVA6 | CVA6 |
| Q1179Y1 | Pan-EV | 29.82 | Negative | CVA6 | CVA6 |
| Q1182Y1 | Pan-EV | 21.51 | Negative | CVA6 | CVA6 |
| Q1183Y1 | Pan-EV | 26.26 | Negative | CVA6 | CVA6 |
| Q1190Y1 | Pan-EV | 34.15 | Negative | CVA6 | CVA6 |
| Q1192Y1 | Pan-EV | 29.28 | Negative | CVA6 | CVA6 |
| Q1191Y1 | Pan-EV | 30.07 | Negative | CVA6 | CVA6 |
| Q1196Y1 | Pan-EV | 31.03 | Negative | CVA6 | CVA6 |
| H1196Y1 | Pan-EV | 28.6 | Negative | CVA6 | CVA6 |
| H1197Y1 | Pan-EV | 31.48 | Negative | CVA6 | CVA6 |
| Q1197Y1 | Pan-EV | 27.34 | Negative | CVA6 | CVA6 |
| Q1201Y1 | Pan-EV | 32.37 | Negative | CVA6 | CVA6 |
| Q1202Y1 | Pan-EV | 32.01 | Negative | CVA6 | CVA6 |
| Q1203Y1 | Pan-EV | 35.78 | Negative | CVA6 | CVA6 |
| H1199Y1 | Pan-EV | 31.78 | Negative | CVA6 | CVA6 |
| H1200Y1 | Pan-EV | 27.33 | Negative | CVA6 | CVA6 |
| H1201Y1 | Pan-EV | 24.42 | Negative | CVA6 | CVA6 |
| H1202Y1 | Pan-EV | 32.95 | Negative | CVA6 | CVA6 |
| H1204Y1 | Pan-EV | 26.77 | Negative | CVA6 | CVA6 |
| H1203Y1 | Pan-EV | 31.63 | Negative | CVA6 | CVA6 |
| Q1204Y1 | Pan-EV | 23.98 | Negative | CVA6 | CVA6 |
| Q1205Y1 | Pan-EV | 32.21 | Negative | CVA6 | CVA6 |
| Q1206Y1 | Pan-EV | 24.28 | Negative | CVA6 | CVA6 |
| Q1207Y1 | Pan-EV | 28.98 | Negative | CVA6 | CVA6 |
| Q1209Y1 | Pan-EV | 29.51 | Negative | CVA6 | CVA6 |
| Q1208Y1 | Pan-EV | 31.8 | Negative | CVA6 | CVA6 |
| Q1210Y1 | Pan-EV | 31.66 | Negative | CVA6 | CVA6 |
| Q1211Y1 | Pan-EV | 25.89 | Negative | CVA6 | CVA6 |
| Q1212Y1 | Pan-EV | 28.78 | Negative | CVA6 | CVA6 |
| Q1214Y1 | Pan-EV | 30.82 | Negative | CVA6 | CVA6 |
| Q1215Y1 | Pan-EV | 31.37 | Negative | CVA6 | CVA6 |
| Q1216Y1 | Pan-EV | 28.18 | Negative | CVA6 | CVA6 |
| Q1218Y1 | Pan-EV | 32.51 | Negative | CVA6 | CVA6 |
| Q1220Y1 | Pan-EV | 28.06 | Negative | CVA6 | CVA6 |
| Q1221Y1 | Pan-EV | 31.25 | Negative | CVA6 | CVA6 |
| Q1222Y1 | Pan-EV | 25.38 | Negative | CVA6 | CVA6 |
| Q1224Y1 | Pan-EV | 32.76 | Negative | CVA6 | CVA6 |
| Q1225Y1 | Pan-EV | 28.97 | Negative | CVA6 | CVA6 |
| Q1227Y1 | Pan-EV | 34.24 | Negative | CVA6 | CVA6 |
| Q1228Y1 | Pan-EV | 34.8 | Negative | CVA6 | CVA6 |
| Q1230Y1 | Pan-EV | 29.17 | Negative | CVA6 | CVA6 |
| Q1234Y1 | Pan-EV | 31.27 | Negative | CVA6 | CVA6 |
| Q1235Y1 | Pan-EV | 32.62 | Negative | CVA6 | CVA6 |
| Q1236Y1 | Pan-EV | 32.12 | Negative | CVA6 | CVA6 |
| Q1238Y1 | Pan-EV | 29.84 | Negative | CVA6 | CVA6 |
| Q1239Y1 | Pan-EV | 28.93 | Negative | CVA6 | CVA6 |
| Q1248Y1 | Pan-EV | 33.08 | Negative | CVA6 | CVA6 |
| Q1249Y1 | Pan-EV | 27.87 | Negative | CVA6 | CVA6 |
| Q1251Y1 | Pan-EV | 30.63 | Negative | CVA6 | CVA6 |
| Q1256Y1 | Pan-EV | 30.78 | Negative | CVA6 | CVA6 |
| Q1257Y1 | Pan-EV | 30.22 | Negative | CVA6 | CVA6 |
| Q1258Y1 | Pan-EV | 32.57 | Negative | CVA6 | CVA6 |
| Q1260Y1 | Pan-EV | 30.96 | Negative | CVA6 | CVA6 |
| H1206Y1 | Pan-EV | 33.53 | Negative | CVA6 | CVA6 |
| H1207Y1 | Pan-EV | 27.51 | Negative | CVA6 | CVA6 |
| H1208Y1 | Pan-EV | 34.75 | Negative | CVA6 | CVA6 |
| H1209Y1 | Pan-EV | 29.27 | Negative | CVA6 | CVA6 |
| H1211Y1 | Pan-EV | 29.03 | Negative | CVA6 | CVA6 |
| H1212Y1 | Pan-EV | 35.94 | Negative | CVA6 | CVA6 |
| H1213Y1 | Pan-EV | 32.26 | Negative | CVA6 | CVA6 |
| H1214Y1 | Pan-EV | 25.81 | Negative | CVA6 | CVA6 |
| H1216Y1 | Pan-EV | 30.96 | Negative | CVA6 | CVA6 |
| H1220Y1 | Pan-EV | 29.12 | Negative | CVA6 | CVA6 |
| H1221Y1 | Pan-EV | 32.5 | Negative | CVA6 | CVA6 |
| H1217Y1 | Pan-EV | 33.48 | Negative | CVA6 | CVA6 |
| H1218Y1 | Pan-EV | 33.16 | Negative | CVA6 | CVA6 |
| H1219Y1 | Pan-EV | 27.3 | Negative | CVA6 | CVA6 |
| H1223Y1 | Pan-EV | 36.04 | Negative | CVA6 | CVA6 |
| H1226Y1 | Pan-EV | 35.24 | Negative | CVA6 | CVA6 |
| H1231Y1 | Pan-EV | 30.2 | Negative | CVA6 | CVA6 |
| H1229Y1 | Pan-EV | 28.72 | Negative | CVA6 | CVA6 |
| H1234Y1 | Pan-EV | 28.9 | Negative | CVA6 | CVA6 |
| H1235Y1 | Pan-EV | 34.38 | Negative | CVA6 | CVA6 |
| H1236Y1 | Pan-EV | 28.35 | Negative | CVA6 | CVA6 |
| H1237Y1 | Pan-EV | 32.91 | Negative | CVA6 | CVA6 |
| H1238Y1 | Pan-EV | 32.42 | Negative | CVA6 | CVA6 |
| H1239Y1 | Pan-EV | 31.75 | Negative | CVA6 | CVA6 |
| H1241Y1 | Pan-EV | 27.18 | Negative | CVA6 | CVA6 |
| H1240Y1 | Pan-EV | 36.48 | Negative | CVA6 | CVA6 |
| H1244Y1 | Pan-EV | 26.75 | Negative | CVA6 | CVA6 |
| H1246Y1 | Pan-EV | 31.29 | Negative | CVA6 | CVA6 |
| H1247Y1 | Pan-EV | 22.96 | Negative | CVA6 | CVA6 |
| H1245Y1 | Pan-EV | 30.46 | Negative | CVA6 | CVA6 |
| H1250Y1 | Pan-EV | 25.81 | Negative | CVA6 | CVA6 |
| H1248Y1 | Pan-EV | 30.71 | Negative | CVA6 | CVA6 |
| H1249Y1 | Pan-EV | 27.95 | Negative | CVA6 | CVA6 |
| H1251Y1 | Pan-EV | 29.73 | Negative | CVA6 | CVA6 |
| H1254Y1 | Pan-EV | 30.19 | Negative | CVA6 | CVA6 |
| H1256Y1 | Pan-EV | 28.13 | Negative | CVA6 | CVA6 |
| H1257Y1 | Pan-EV | 29.55 | Negative | CVA6 | CVA6 |
| H1258Y1 | Pan-EV | 27.72 | Negative | CVA6 | CVA6 |
| H1262Y1 | Pan-EV | 26.16 | Negative | CVA6 | CVA6 |
| H1260Y1 | Pan-EV | 32.96 | Negative | CVA6 | CVA6 |
| H1263Y1 | Pan-EV | 23.29 | Negative | CVA6 | CVA6 |
| H1265Y1 | Pan-EV | 22.62 | Negative | CVA6 | CVA6 |
| H1266Y1 | Pan-EV | 24.33 | Negative | CVA6 | CVA6 |
| H1267Y1 | Pan-EV | 31.95 | Negative | CVA6 | CVA6 |
| H1268Y1 | Pan-EV | 27.91 | Negative | CVA6 | CVA6 |
| H1269Y1 | Pan-EV | 29.66 | Negative | CVA6 | CVA6 |
| H1270Y1 | Pan-EV | 25.05 | Negative | CVA6 | CVA6 |
| H1271Y1 | Pan-EV | 33.73 | Negative | CVA6 | CVA6 |
| H1272Y1 | Pan-EV | 23.14 | Negative | CVA6 | CVA6 |
| H1283Y1 | Pan-EV | 24.79 | Negative | CVA6 | CVA6 |
| H1273Y1 | Pan-EV | 20.18 | Negative | CVA6 | CVA6 |
| H1274Y1 | Pan-EV | 27.25 | Negative | CVA6 | CVA6 |
| H1284Y1 | Pan-EV | 31.85 | Negative | CVA6 | CVA6 |
| H1275Y1 | Pan-EV | 25.23 | Negative | CVA6 | CVA6 |
| H1276Y1 | Pan-EV | 27.84 | Negative | CVA6 | CVA6 |
| H1285Y1 | Pan-EV | 28.09 | Negative | CVA6 | CVA6 |
| H1277Y1 | Pan-EV | 26.53 | Negative | CVA6 | CVA6 |
| H1278Y1 | Pan-EV | 29.49 | Negative | CVA6 | CVA6 |
| H1279Y1 | Pan-EV | 33.85 | Negative | CVA6 | CVA6 |
| H1287Y1 | Pan-EV | 25.01 | Negative | CVA6 | CVA6 |
| H1280Y1 | Pan-EV | 25.69 | Negative | CVA6 | CVA6 |
| H1281Y1 | Pan-EV | 29.36 | Negative | CVA6 | CVA6 |
| H1289Y1 | Pan-EV | 26.72 | Negative | CVA6 | CVA6 |
| H1290Y1 | Pan-EV | 22.99 | Negative | CVA6 | CVA6 |
| H1288Y1 | Pan-EV | 22.07 | Negative | CVA6 | CVA6 |
| H1298Y1 | Pan-EV | 23.63 | Negative | CVA6 | CVA6 |
| H1297Y1 | Pan-EV | 25.27 | Negative | CVA6 | CVA6 |
| H1296Y1 | Pan-EV | 24.55 | Negative | CVA6 | CVA6 |
| H1295Y1 | Pan-EV | 29.39 | Negative | CVA6 | CVA6 |
| H1292Y1 | Pan-EV | 29.55 | Negative | CVA6 | CVA6 |
| H1291Y1 | Pan-EV | 24.99 | Negative | CVA6 | CVA6 |
| H1293Y1 | Pan-EV | 21.04 | Negative | CVA6 | CVA6 |
| H1300Y1 | Pan-EV | 27.39 | Negative | CVA6 | CVA6 |
| H1302Y1 | Pan-EV | 25.94 | Negative | CVA6 | CVA6 |
| H1304Y1 | Pan-EV | 26.87 | Negative | CVA6 | CVA6 |
| H1308Y1 | Pan-EV | 29.78 | Negative | CVA6 | CVA6 |
| H1307Y1 | Pan-EV | 28.11 | Negative | CVA6 | CVA6 |
| H1306Y1 | Pan-EV | 25.81 | Negative | CVA6 | CVA6 |
| H1305Y1 | Pan-EV | 32.82 | Negative | CVA6 | CVA6 |
| H1315Y1 | Pan-EV | 27.42 | Negative | CVA6 | CVA6 |
| H1316Y1 | Pan-EV | 25.44 | Negative | CVA6 | CVA6 |
| H1311Y1 | Pan-EV | 23.33 | Negative | CVA6 | CVA6 |
| H1317Y1 | Pan-EV | 24.27 | Negative | CVA6 | CVA6 |
| H1318Y1 | Pan-EV | 24.1 | Negative | CVA6 | CVA6 |
| H1159Y1 | Pan-EV | 33.78 | Negative | CVA6 | CVA6 |
| Q1149Y1 | Pan-EV | 29.28 | Negative | CVA6 | CVA6 |
| H1325Y1 | Pan-EV | 28.13 | Negative | CVA6 | CVA6 |
| H1324Y1 | Pan-EV | 27.13 | Negative | CVA6 | CVA6 |
| H1322Y1 | Pan-EV | 26.92 | Negative | CVA6 | CVA6 |
| H1320Y1 | Pan-EV | 26.14 | Negative | CVA6 | CVA6 |
| H1326Y1 | Pan-EV | 35.05 | Negative | CVA6 | CVA6 |
| H1327Y1 | Pan-EV | 26.94 | Negative | CVA6 | CVA6 |
| H1328Y1 | Pan-EV | 26.87 | Negative | CVA6 | CVA6 |
| H1331Y1 | Pan-EV | 21.72 | Negative | CVA6 | CVA6 |
| H1329Y1 | Pan-EV | 28.46 | Negative | CVA6 | CVA6 |
| H1330Y1 | Pan-EV | 26.24 | Negative | CVA6 | CVA6 |
| H1334Y1 | Pan-EV | 30.17 | Negative | CVA6 | CVA6 |
| H1339Y1 | Pan-EV | 27.35 | Negative | CVA6 | CVA6 |
| H1338Y1 | Pan-EV | 24.57 | Negative | CVA6 | CVA6 |
| H1340Y1 | Pan-EV | 27.78 | Negative | CVA6 | CVA6 |
| H1341Y1 | Pan-EV | 27.32 | Negative | CVA6 | CVA6 |
| H1342Y1 | Pan-EV | 22.71 | Negative | CVA6 | CVA6 |
| H1344Y1 | Pan-EV | 31.52 | Negative | CVA6 | CVA6 |
| H1345Y1 | Pan-EV | 28.26 | Negative | CVA6 | CVA6 |
| H1347Y1 | Pan-EV | 31.64 | Negative | CVA6 | CVA6 |
| H1142Y1 | Pan-EV | 19.54 | Negative | CVA8 | CVA8 |
| H1009Y1 | Pan-EV | 36.41 | Negative | EV-A71 | EV-A71 |
| H1041Y1 | Pan-EV | 26.39 | Negative | EV-A71 | EV-A71 |
| Q1084Y1 | Pan-EV | 29.49 | Negative | EV-A71 | EV-A71 |
| Q1121Y1 | Pan-EV | 29.19 | Negative | EV-A71 | EV-A71 |
| H1152Y1 | Pan-EV | 28.4 | Negative | EV-A71 | EV-A71 |
| H1184Y1 | Pan-EV | 29.77 | Negative | EV-A71 | EV-A71 |
| H1188Y1 | Pan-EV | 29.83 | Negative | EV-A71 | EV-A71 |
| H1205Y1 | Pan-EV | 34.76 | Negative | EV-A71 | EV-A71 |
| H1261Y1 | Pan-EV | 24.76 | Negative | EV-A71 | EV-A71 |
| H1025Y1 | Pan-EV | 33.28 | Negative | HRV A | HRV A |
| Q1091Y1 | Pan-EV | 30.8 | Negative | HRV A | HRV A |
| H1028Y1 | Pan-EV | 30.86 | Negative | HRV C | HRV C |
| Q1237Y1 | Pan-EV | 30.46 | Negative | HRV C | HRV C |
| Q1003Y1 | Pan-EV | 34.47 | Negative | Negative | Pan-EV |
| Q1002Y1 | Pan-EV | 25.85 | Negative | Negative | Pan-EV |
| H1004Y1 | Pan-EV | 32.56 | Negative | Negative | Pan-EV |
| Q1006Y1 | Pan-EV | 29.65 | Negative | Negative | Pan-EV |
| Q1010Y1 | Pan-EV | 30.36 | Negative | Negative | Pan-EV |
| H1017Y1 | Pan-EV | 30.56 | Negative | Negative | Pan-EV |
| Q1013Y1 | Pan-EV | 27.35 | Negative | Negative | Pan-EV |
| Q1020Y1 | Pan-EV | 30.21 | Negative | Negative | Pan-EV |
| Q1021Y1 | Pan-EV | 31.34 | Negative | Negative | Pan-EV |
| H1030Y1 | Pan-EV | 29.34 | Negative | Negative | Pan-EV |
| Q1025Y1 | Pan-EV | 33.61 | Negative | Negative | Pan-EV |
| Q1034Y1 | Pan-EV | 36.09 | Negative | Negative | Pan-EV |
| Q1048Y1 | Pan-EV | 30.27 | Negative | Negative | Pan-EV |
| Q1028Y1 | Pan-EV | 31.3 | Negative | Negative | Pan-EV |
| H1073Y1 | Pan-EV | 31.98 | Negative | Negative | Pan-EV |
| Q1080Y1 | Pan-EV | 32.06 | Negative | Negative | Pan-EV |
| Q1079Y1 | Pan-EV | 31.94 | Negative | Negative | Pan-EV |
| Q1085Y1 | Pan-EV | 32.57 | Negative | Negative | Pan-EV |
| H1096Y1 | Pan-EV | 31.81 | Negative | Negative | Pan-EV |
| Q1102Y1 | Pan-EV | 30.84 | Negative | Negative | Pan-EV |
| Q1118Y1 | Pan-EV | 29.66 | Negative | Negative | Pan-EV |
| Q1129Y1 | Pan-EV | 30.06 | Negative | Negative | Pan-EV |
| Q1130Y1 | Pan-EV | 29.25 | Negative | Negative | Pan-EV |
| H1154Y1 | Pan-EV | 24.42 | Negative | Negative | Pan-EV |
| H1167Y1 | Pan-EV | 37.06 | Negative | Negative | Pan-EV |
| H1161Y1 | Pan-EV | 30.35 | Negative | Negative | Pan-EV |
| H1180Y1 | Pan-EV | 27.62 | Negative | Negative | Pan-EV |
| H1179Y1 | Pan-EV | 29.67 | Negative | Negative | Pan-EV |
| Q1160Y1 | Pan-EV | 34.29 | Negative | Negative | Pan-EV |
| Q1165Y1 | Pan-EV | 32.11 | Negative | Negative | Pan-EV |
| Q1171Y1 | Pan-EV | 30.03 | Negative | Negative | Pan-EV |
| Q1175Y1 | Pan-EV | 32.7 | Negative | Negative | Pan-EV |
| Q1189Y1 | Pan-EV | 30.6 | Negative | Negative | Pan-EV |
| Q1187Y1 | Pan-EV | 30.59 | Negative | Negative | Pan-EV |
| Q1194Y1 | Pan-EV | 30.57 | Negative | Negative | Pan-EV |
| Q1219Y1 | Pan-EV | 37.65 | Negative | Negative | Pan-EV |
| Q1223Y1 | Pan-EV | 34.21 | Negative | Negative | Pan-EV |
| Q1229Y1 | Pan-EV | 33.33 | Negative | Negative | Pan-EV |
| Q1231Y1 | Pan-EV | 33.22 | Negative | Negative | Pan-EV |
| Q1233Y1 | Pan-EV | 34.36 | Negative | Negative | Pan-EV |
| Q1243Y1 | Pan-EV | 29.17 | Negative | Negative | Pan-EV |
| Q1245Y1 | Pan-EV | 30.15 | Negative | Negative | Pan-EV |
| Q1246Y1 | Pan-EV | 29.55 | Negative | Negative | Pan-EV |
| Q1252Y1 | Pan-EV | 31.62 | Negative | Negative | Pan-EV |
| Q1254Y1 | Pan-EV | 33.01 | Negative | Negative | Pan-EV |
| H1210Y1 | Pan-EV | 30.39 | Negative | Negative | Pan-EV |
| H1215Y1 | Pan-EV | 27.91 | Negative | Negative | Pan-EV |
| H1222Y1 | Pan-EV | 29.73 | Negative | Negative | Pan-EV |
| H1224Y1 | Pan-EV | 30.01 | Negative | Negative | Pan-EV |
| H1225Y1 | Pan-EV | 29.72 | Negative | Negative | Pan-EV |
| H1227Y1 | Pan-EV | 29.17 | Negative | Negative | Pan-EV |
| H1228Y1 | Pan-EV | 28.75 | Negative | Negative | Pan-EV |
| H1232Y1 | Pan-EV | 31.58 | Negative | Negative | Pan-EV |
| H1233Y1 | Pan-EV | 31.48 | Negative | Negative | Pan-EV |
| H1243Y1 | Pan-EV | 37.27 | Negative | Negative | Pan-EV |
| H1242Y1 | Pan-EV | 30.24 | Negative | Negative | Pan-EV |
| H1253Y1 | Pan-EV | 26.3 | Negative | Negative | Pan-EV |
| H1255Y1 | Pan-EV | 34.16 | Negative | Negative | Pan-EV |
| H1259Y1 | Pan-EV | 34.13 | Negative | Negative | Pan-EV |
| H1282Y1 | Pan-EV | 35.27 | Negative | Negative | Pan-EV |
| H1160Y1 | Pan-EV | 29.87 | Negative | Negative | Pan-EV |
| Q1148Y1 | Pan-EV | 37.39 | Negative | Negative | Pan-EV |
| H1336Y1 | Pan-EV | 29.49 | Negative | Negative | Pan-EV |

Note: “– “ indicates that a test was not conducted. Green indicates the sample was typed by real-time RT-PCR; orange indicates the sample was typed by VP1-based nested RT-PCR; purple indicates the sample was typed by VP4-VP2-based nested RT-PCR; blue indicates the sample was an untyped enterovirus.
